# Effort, timing and choice: a refined rodent effort choice task with excellent sensitivity to dopamine modulators

**DOI:** 10.1038/s41386-026-02453-8

**Published:** 2026-06-10

**Authors:** Suellen Almeida-Correa, Fynn-Sebastian Wesemann, Sarah Diez, Mary Jane Maynard, Lucas Yebra Garcia, Patrizia Voehringer, Boris Ferger, Michael Becker, Carsten T. Wotjak, Johann du Hoffmann

**Affiliations:** 1 Neuroscience and Mental Health Discovery Research Department, Boehringer Ingelheim Pharma GmbH, Biberach, Germany; 2 Drug Metabolism and Pharmacokinetics, Boehringer Ingelheim Pharma GmbH, Biberach, Germany; 3 Faculty of Biology, Medicine and Health, University of Manchester, Manchester, United Kingdom; 4 Department of Brain Sciences, Faculty of Medicine, Imperial College London, London, United Kingdom; 5 Ulm University Medical Center, Institute of Experimental and Clinical Pharmacology, Toxicology and Pharmacology of Natural Products, Ulm, Germany

**Keywords:** Motivation, Reward, Pharmacology

## Abstract

Motivational deficits and anhedonia are core features of psychiatric disorders such as depression, schizophrenia and addiction, yet current treatments rarely improve these symptoms. Progress towards targeted therapeutics requires preclinical tasks that reliably capture pharmacological modulation of relevant neural circuits. Here, we developed a modified effort-related choice task (mERC) that integrates high effort demands, extended intertrial intervals and choice, to enable robust bidirectional sensitivity to systemic pharmacology. Unlike standard paradigms, mERC detects both impairing and enhancing effects of dopamine modulators within a single task and reveals drug-sensitive anticipatory responses during intertrial intervals—a behavioral feature linked to interval timing and motivational vigor. Using neurochemical and photometric analyses, we show that mERC performance is associated with behavioral signatures consistent with dopaminergic signaling across different timescales and is potentially constrained by a bounded dynamic range beyond which behavioral modulation is lost. Species differences in drug efficacy map onto baseline dopamine tone and depletion dynamics, underscoring the interactions between task structure and neurochemical state. Together, these findings establish mERC as a stable and sensitive preclinical assay for enhancing our understanding of effort-based decision-making and for screening novel therapeutics targeting motivational dysfunction.

## Introduction

The ability to detect rewards and initiate motor actions to obtain them is highly adaptive and disruptions in these processes are core symptoms of neuropsychiatric diseases [1–4] such as depression [5–7] and addiction [8]. Dopamine (DA) is integral to the anticipation [9, 10] and realization of rewards [11], shaping motivated behavior through its influence on reward processing [12], prediction [13], learning [14, 15], and motivational vigor [16–18]. Impaired DA signaling—whether by pharmacological depletion [19–21], D1 or D2 receptor antagonists [22], or lesions [23], or other disruptions— reduces willingness to exert effort to obtain rewards and diminishes responding to reward-predictive cues, modeling key aspects of human motivational impairment [24, 25].

Effort-related choice (ERC) tasks, in which rodents choose between exerting effort for a preferred reward or consuming freely available lab chow [26–29] are widely used to evaluate motivation and test candidate therapeutics. ERC tasks have demonstrated translational value [30], and human ERC-related tasks [31–37] have shown that, like in rat, dopamine is a key neurotransmitter disrupted in depression [38] and the negative symptoms of schizophrenia [2, 39]. However, standard preclinical ERC tasks often fail to detect both decreases and increases in motivation within the same behavioral context, which is critical for understanding the dose–response relationship and the therapeutic potential of novel pharmacotherapies.

Current preclinical approaches typically pair ERC tasks with dopamine-impairing drugs such as the VMAT-2 inhibitor tetrabenazine and then test whether putative pro-motivational drugs reverse these deficits [20, 40]. Although useful, this strategy introduces complexities like drug-drug interactions and may obscure pro-motivational effects of compounds acting upstream of DA release [41]. Thus, a relevant task that detects motivational enhancement without requiring pharmacological challenge would simplify and provide a more solid foundation for preclinical testing of novel pharmacotherapies targeting motivational deficits.

Here, we developed a modified ERC task (mERC) designed for robust bidirectional sensitivity to systemic pharmacology. By increasing effort requirements, adding a 30-second intertrial interval (ITI) and including a cue signaling reward availability, we created a highly responsive behavioral assay with strong sensitivity to changes in dopamine tone. Our results demonstrate that mERC is a powerful and versatile preclinical tool for identifying and characterizing potential drugs to treat motivational deficits in psychiatric disorders.

## Methods

### Animals

Male rats and mice were group housed (2–4/cage) on a 12-h light/dark cycle. Experiments were conducted during the light phase. After acclimation, animals were food-restricted to ~90% free-feeding weight for behavioral testing. All procedures were approved by the Regierungspraesidium Tübingen and complied with AAALAC and EU Directive 2010/63/EU. Details are listed in Supplementary Table S1.

### Operant chambers

Rat experiments used Med Associates chambers with a lever, pellet dispenser and photobeam‑equipped reward receptacle. Mouse experiments used Bussey–Saksida touchscreen chambers [28] delivering 10% liquid sucrose via pump. Behavioral events were recorded at 1-ms resolution. See Supplemental Materials for details.

### Rat effort-related choice (ERC) tasks

**Fig. 1:**
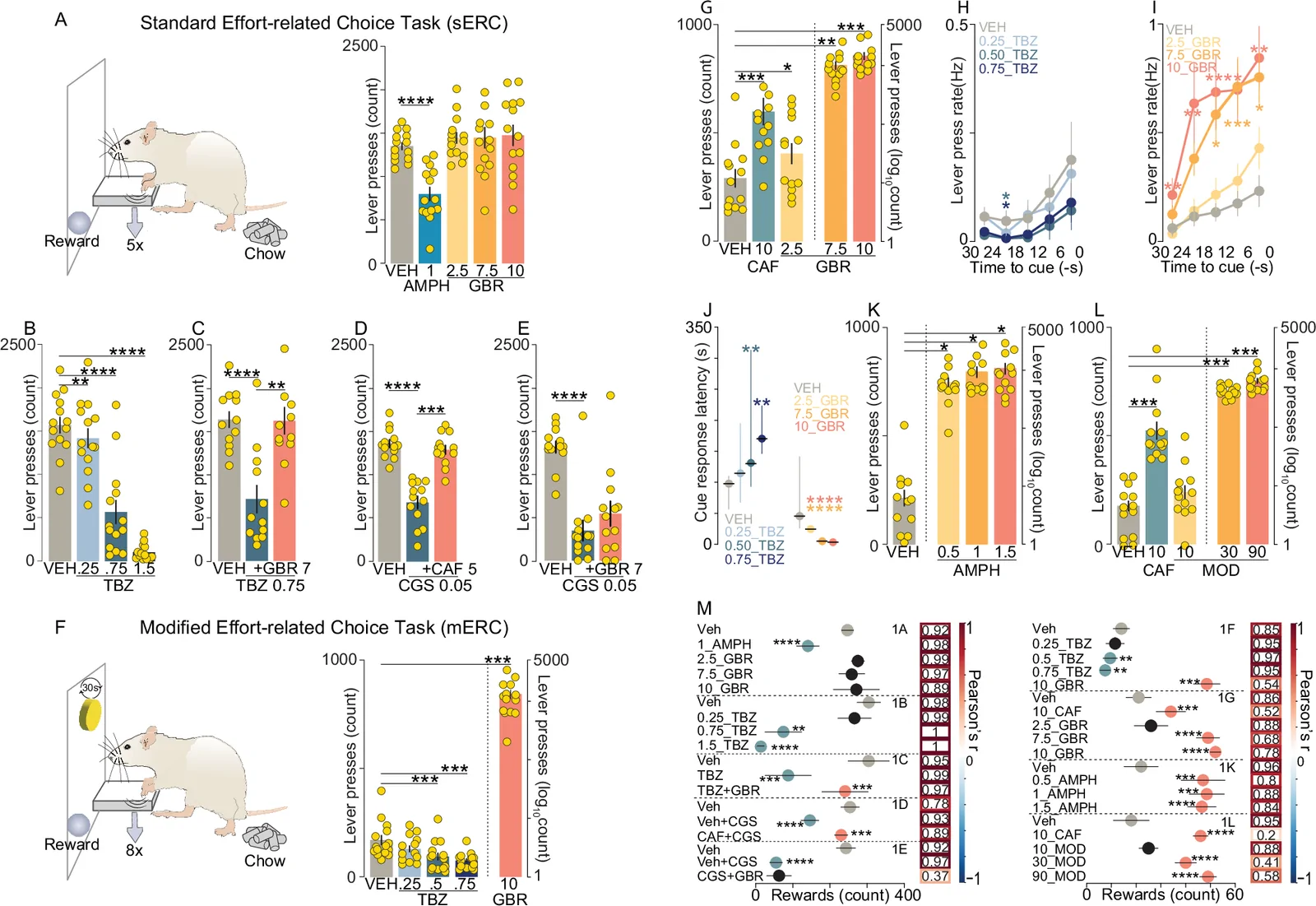
The modified effort-related choice task (mERC) enables bidirectional dopaminergic modulation of reward seeking and effortful responding in rats. Standard (**A**–**E**, sERC) vs. modified effort-related choice (**F**–**L**, mERC) in rats. **A** sERC schematic (left) and mean lever presses in male SD rats (*n* = 14) treated with vehicle or the DAT inhibitors amphetamine (AMPH) or GBR‑12909 (GBR; mg/kg). Mean lever presses following **B** TBZ dose–response, **C** TBZ alone or with GBR, **D** CGS‑21680 (CGS) alone or with caffeine (CAF), and **E** CGS with GBR (*n* = 13). **F** mERC schematic (left) and mean lever presses after vehicle, TBZ, or GBR (*n* = 14). **G** Lever presses after caffeine or GBR (*n* = 13). Binned ITI lever‑press rates for (**H**) TBZ (from **F**) and (**I**) GBR (from **G**). **J** Median response latencies with interquartile range from (**F**) and (**G**). **K** Mean lever presses after vehicle or AMPH dose–response (*n* = 12). **L** Mean lever presses after caffeine or modafinil (MOD) dose–response (*n* = 13). **M** Median rewards (inner quartiles) and Pearson’s r correlations between rewards and lever presses for (**A**–**G**) and (**K**–**L**). Statistics in Supplementary Tables S5–S6.

Rats were trained on fixed-ratio FR1 and FR5 lever pressing for sucrose pellets (>1000 presses/30 min criterion). In standard ERC (sERC), rats chose between FR5 responding and freely available chow (Fig. 1A–E). In modified ERC (mERC; Fig. 1F–L), effort increased to FR8 with chow available and each trial was preceded by a 30-s ITI. A cue light above the lever signaled ITI offset and was extinguished upon reward delivery, while the lever remained extended throughout the session. During extinction, rewards were omitted (Fig. 3A). Pre-feeding in Fig. 3C matched typical mERC chow intake, which was measured post-session (Supplementary Fig. S1). See Supplementary Table S2 and Supplementary Fig. S2 for details.

### Mouse effort-related choice (ERC) tasks

Mice were initially trained to collect 10% sucrose (60 µL; reward) and then to touch a 4 × 4 cm white square (FR1) within a touchscreen mask aperture for reward. The cue was initially presented at the bottom of the screen and later raised to 5.5 cm to increase effort; the aperture went black after rewarded responses and during ITIs. After reaching criteria (>40 rewards for 2 consecutive days), mice performed ERC with a choice between touchscreen responding and freely available chow.

**Fig. 2:**
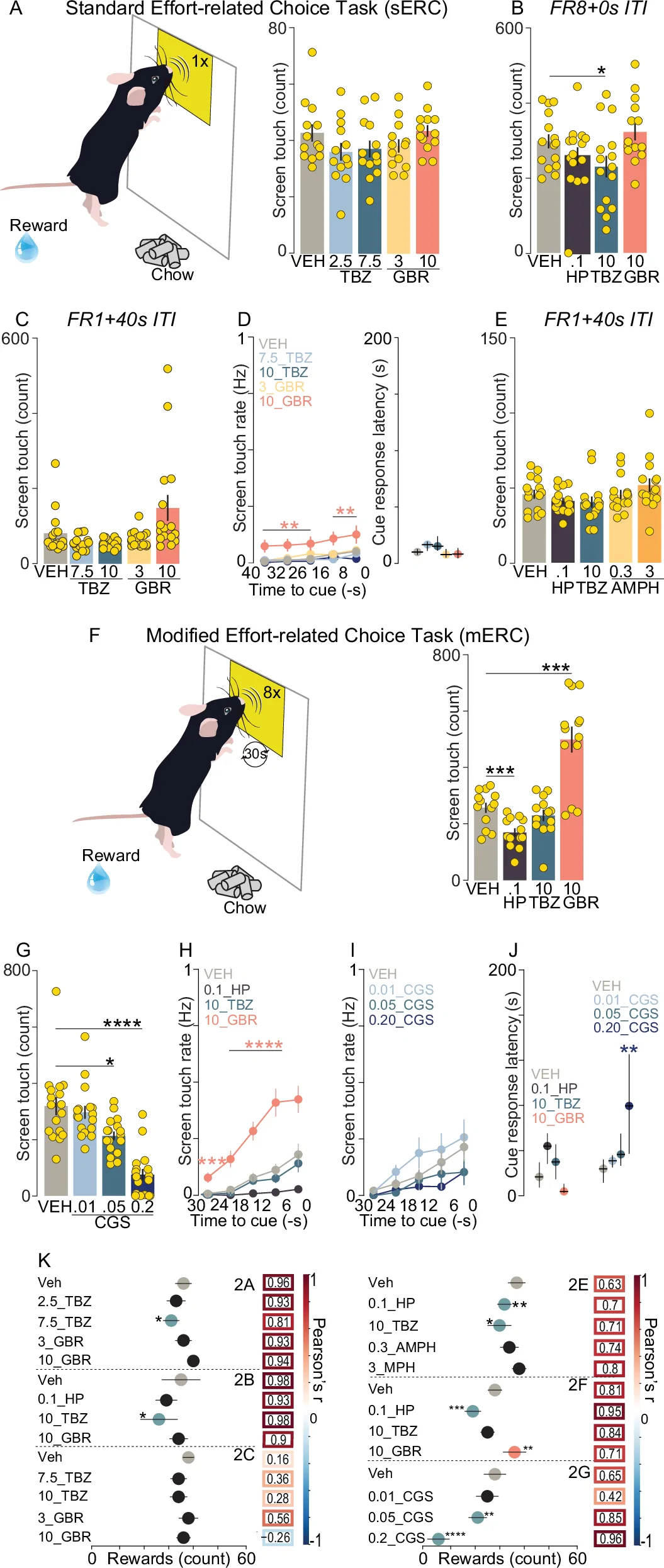
Task structure determines dopaminergic sensitivity in mouse effort-related choice, enabling bidirectional modulation under mERC conditions. Standard (**A**–**E**, sERC) vs. modified effort-related choice (**F**–**J**, mERC) in mice. **A** sERC schematic (left) and mean screen touches in male C57BL/6 mice treated with vehicle, tetrabenazine (TBZ), or GBR‑12909 (GBR; *n* = 13; mg/kg). **B** ERC FR8: screen touches after vehicle, haloperidol (HP), TBZ, or GBR (*n* = 15). **C** ERC FR1 + 40 s ITI: same drugs as in (**A**). **D** Binned ITI screen‑touch rates and cue response latencies from (**C**). **E** ERC FR1 + 40 s ITI: vehicle, HP, TBZ, amphetamine (AMPH), or methylphenidate (MPH). **F** mERC schematic (left) and mean screen touches after vehicle, HP, TBZ, or GBR (*n* = 13). **G** Vehicle and CGS‑21680 (CGS) dose–response (*n* = 17). Binned ITI screen‑touch rates from (**H**) **F** and (**I**) **G**. **J** Median response latencies (IQR) from (**F**) and (**G**). **K** Median rewards (inner quartiles) and Pearson’s r correlations between rewards and screen touches for (**A**–**C**) and (**E**–**G**). Statistics in Supplementary Tables S7, S8.

In mERC (Fig. 2F–J), the requirement was FR8 with a 30-second intertrial interval (ITI) in 45 min sessions and cue control like the rat task. See Supplementary Table S3 and Supplementary Fig. S2 for details.

### Drugs

Drugs were freshly prepared and administered systemically twice weekly in a crossover design. Compounds included: amphetamine (AMPH); caffeine (CAF); CGS-21680 (CGS); GBR-12909 (GBR); haloperidol (HP); modafinil (MOD); and tetrabenazine (TBZ). Formulation and administration details are listed in Supplementary Table S4. Behavioral doses were selected from dose–response or published data to define an ED50; dopamine measurements used behaviorally relevant doses, including the ED50 unless method sensitivity required higher doses.

### Microdialysis

As previously described [42], guide cannulae were implanted above rat dorsal striatum and nucleus accumbens (NAc). After 1-week, probes were inserted and perfused with aCSF; 1μL dialysate samples were collected every 20 min following tetrabenazine or vehicle and then GBR-12909 or vehicle. Dopamine concentrations were quantified by LC-MS/MS. Probe placement was confirmed histologically (see Supplemental Methods).

### Fiber photometry

Rats received NAc injections of AAV1/2-hSyn-GRAB_DA2m(DA4.4) [43] were trained on mERC and implanted with optic fibers. Dopamine signals were recorded during vehicle, GBR-12909 or TBZ sessions in a crossover design. Viral expression and fiber placement were verified histologically. See Supplemental Methods for details.

### Matrix-assisted laser desorption ionization mass spectrometry imaging (MALDI-MSI)

Rats and mice received tetrabenazine or vehicle 90 minutes before euthanasia. Brains were frozen, sectioned (12 µm coronal slices), mounted on conductive glass slides and spray-coated with FMP-10 matrix. MALDI-MSI quantified peak intensity of derivatized dopamine ([DA + 2[FMP-10]-CH3]+: m/z 674.2807). Hot spot removal was applied. Additional protocol details are provided in Supplemental Methods.

### Data analysis

Data were analyzed using custom R scripts (R Core Team, 2013) or GraphPad Prism (v.10.4.1). Paired/unpaired *t*-tests or ANOVA with appropriate post hoc tests (Tukey, Dunnett or Sidak) were used for statistics. Significance was set at α = 0.05. (*p < 0.05, **p < 0.01, ***p < 0.001, ****p < 0.0001). Comprehensive statistics are reported in Supplementary Tables S5–S11.

## Results

### Pharmacology of effort-based decision-making in rats

In the rat standard Effort-Related Choice Task (sERC [22, 26]) we tested two dopamine transporter (DAT) inhibitors: amphetamine, which blocks reuptake and promotes dopamine release, and GBR-12909, which blocks reuptake without altering release. Amphetamine reduced lever pressing and reward acquisition (Fig. 1A, M), while GBR-12909 had no effect. Consistent with previous reports [20, 21, 44, 45], tetrabenazine (TBZ) and the A2A agonist CGS-21680 (CGS), impaired sERC performance (Fig. 1B–E). These impairments were reversed by GBR-12909 and caffeine, respectively—but only when the reversal matched the impairer’s mechanism of action (e.g., GBR-12909 did not reverse CGS effects; Fig. 1E). Chow intake increased with high-dose TBZ but decreased when TBZ was combined with GBR, or when CGS was combined with caffeine or GBR (Supplementary Fig. S1; Supplementary Table S5). Thus, sERC showed limited sensitivity to dopamine-enhancing drugs: except when paired with a suitable challenge.

To enhance pharmacological sensitivity, we modified task structure by: (1) increasing effort demands from FR5 to FR8, (2) introducing a fixed 30 s intertrial interval (ITI), and (3) adding a light cue above the lever to signal trial onset—features previously shown to increase dopamine-dependency of behavioral tasks [18, 46–48]. Under these conditions (mERC), TBZ impaired behavior (Fig. 1F, M), whereas GBR-12909, amphetamine, and modafinil produced robust dose-dependent increases in lever pressing and rewards (Fig. 1G, K, L). Notably, amphetamine produced opposite effects in sERC versus mERC (Fig. 1A, K), indicating task‑dependent differences in underlying neural circuit engagement. Caffeine also enhanced responding, comparable to dopamine modulators (Fig. 1G, L). Chow intake decreased following GBR, amphetamine and modafinil; while TBZ produced no detectable change (Supplementary Fig. S1; Supplementary Table S5).

### Anticipatory responding and latency effects in rat mERC

mERC showed pronounced latency effects of dopamine modulators: tetrabenazine increased response latency, whereas GBR-12909 decreased it (Fig. 1J). To examine these effects, we quantified lever pressing across the 30 s ITI. Vehicle-treated rats showed clear anticipatory ramping, with press rates rising as cue onset approached (gray trace, Fig. 1H). TBZ dose-dependently suppressed ITI pressing, although the overall ramping pattern remained intact. ANOVA confirmed significant TBZ effects on ITI press rates (Supplementary Table S6), though bin-wise comparisons were modest due to variability and low absolute press rates.

In contrast, GBR-12909 markedly increased ITI pressing (Fig. 1I), with enhanced ramping evident as early as 18 s prior to cue onset. These anticipatory responses contributed to improved overall performance: reduced response latency permitted completion of more trials within the fixed 30-min session, thereby reducing opportunity cost and increasing reward (Fig. 1M), an effect driven by anticipatory lever pressing as the ITI elapsed.

### Dopamine sensitivity in mouse ERC also depends on task structure

A FR1 mouse touchscreen-based ERC (sERC [27]) showed minimal sensitivity to dopamine modulators. Neither tetrabenazine nor GBR-12909 changed the number of screen touches (Fig. 2A), while TBZ produced only modest reductions in rewards earned (Fig. 2K) and chow intake (Supplementary Fig. S1; Supplementary Table S7).

To investigate this weak DA dependency, we systematically tested task parameters previously optimized in rats (see Fig. 1F–L). Increasing effort to FR8 produced mild impairments with high-dose tetrabenazine (10 mg/kg) or haloperidol (0.1 mg/kg), with no effect of GBR-12909 (Fig. 2B, K) beyond decreased chow intake (Supplementary Fig. S1; Supplementary Table S7), indicating that increased effort alone was insufficient to confer strong dopamine sensitivity in mouse sERC

Next, we introduced a 40 s intertrial interval (ITI) while maintaining an FR1 schedule. Under these conditions, tetrabenazine, haloperidol and amphetamine had no effect (Fig. 2C, E), while GBR-12909 elicited a modest increase in screen touches (Fig. 2C), which was uncoupled from rewards earned (Fig. 2K, Pearson’s r). Despite the long ITI, vehicle-treated mice displayed no anticipatory ramping or latency effects (Fig. 2D), and GBR-12909 elicited a small increase in responding. These findings indicated that extending the ITI alone is insufficient to confer dopamine sensitivity in mouse sERC.

### Mouse mERC confers bidirectional dopamine sensitivity

Based on the rat mERC design, we combined FR8 with a fixed 30 s ITI in mice. Under these conditions, haloperidol strongly reduced screen touches and rewards (Fig. 2F, K), while GBR-12909 significantly increased both measures while decreasing chow intake (Supplementary Fig. S1; Supplementary Table S8). Notably, even very high-dose tetrabenazine had no effect. This bidirectional sensitivity in mouse mERC closely matched that observed in rat mERC and was further validated with CGS-21680, which dose-dependently impaired performance (Fig. 2G) and increased chow intake (Supplementary Fig. S1; Supplementary Table S8), consistent with prior rat findings (Fig. 1D, E and refs. [40, 45]).

In mERC, vehicle-treated mice (as in rats) showed ramping lever press rates that were modulated by dopamine-targeting drugs and accompanied by corresponding changes in response latencies: impairing DA signaling increased response latency, while enhancing it shortened latency (Fig. 2H–J). Notably, in mERC, drug effects on screen touches and rewards were tightly correlated (Fig. 2K), whereas under FR8 or long ITI alone (Fig. 2B, C, K), these measures dissociated, with drug effects emerging on only one parameter or the other (see Supplementary Tables S7, S8 for statistics).

### Outcome sensitivity and choice constrain dopaminergic modulation in mERC

**Fig. 3:**
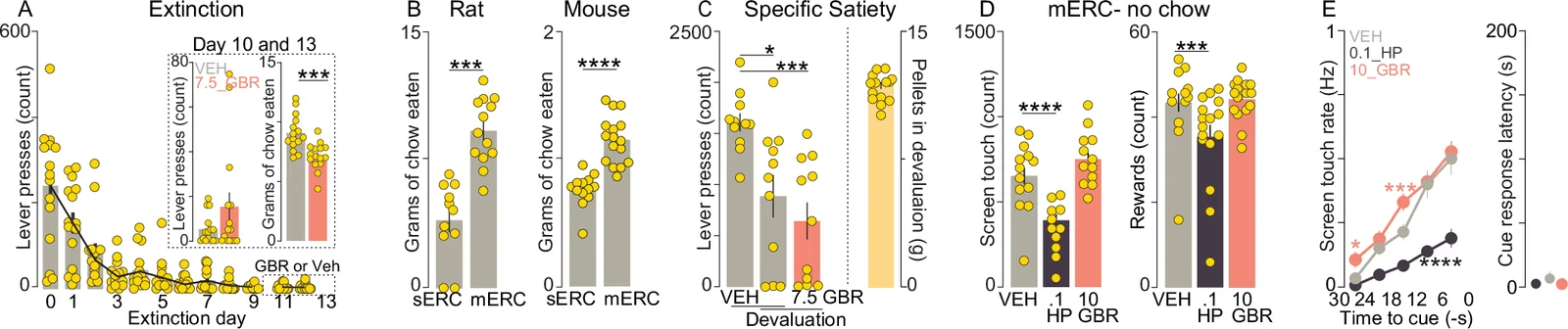
Effects of elevated dopamine on effort-related choice are contingent on reward and choice context. **A** Lever presses during baseline (day 0) and extinction (days 1–9) in rat mERC (*n* = 15). *Inset:* Lever presses and chow intake (g) after vehicle or GBR-12909 (GBR; mg/kg) on test days 10 and 13. **B** Chow intake (g) in sERC- and mERC-trained rats (*n* = 11–13) and mice (*n* = 13–16). **C** Lever presses after vehicle or GBR during a specific-satiety devaluation test and reward pellets consumed (g) during the devaluation phase. **D** mERC without chow choice: screen touches and rewards earned by mice treated with vehicle, haloperidol (HP), or GBR-12909 (*n* = 15). **E** Binned ITI screen-touch rates and cue response latencies (right axis) from (**D**). Statistics in Supplementary Table S9.

Goal-directed and habitual behaviors depend on partially dissociable neural circuits and mechanisms, with dopamine contributing to both [49–55]. A defining feature of goal-directed, unlike habitual, behavior is sensitivity to extinction and action-outcome devaluation [56–59]. To test whether mERC behavior remains sensitive to reward omission—and whether positive dopamine modulation in mERC promotes outcome-independent responding—we administered GBR-12909 to rats after 9 days of extinction training, when lever presses no longer produced reward. Under these conditions, lever responding declined sharply over the first two days, stabilized near zero by day 3, and remained very low for at least a week, consistent with rapid flexible adaptation to updated action-outcome contingencies (Fig. 3A).

To assess whether dopamine enhancement could reinstate responding, rats received GBR-12909 or vehicle in a within-subject crossover design. GBR-12909 had no effect on lever pressing (Fig. 3A, inset), indicating that elevated dopamine alone in mERC is insufficient to reinstate reward seeking following extinction. Thus, mERC behavior remains sensitive to reward omission, consistent with flexible outcome-dependent control. Notably, GBR-treated rats consumed less chow than controls (Fig. 3A, inset and Supplementary Fig. S1), indicating behavioral effects without increasing lever-directed responding.

In mERC, rats and mice consumed significantly more free chow than in sERC (Fig. 3B, Supplementary Fig. S1) raising the possibility that higher chow intake suppresses baseline lever responding, potentially revealing drug effects by removing a response ceiling. To test this, sERC-trained rats were pre-fed an amount of reward pellets matched in weight to the free chow consumed in mERC (Fig. 3C). Pre-feeding reduced lever pressing, consistent with goal-directed sensitivity to outcome devaluation; however, GBR-12909 still failed to restore responding. These findings indicate that increased chow intake does not explain the enhanced dopaminergic sensitivity observed in mERC.

To further evaluate chow’s role, we trained mice on touchscreen mERC and removed chow on the test session (Fig. 3D). Haloperidol still impaired performance, but GBR-12909 no longer enhanced it. Thus, the presence of free chow is critical for revealing bidirectional drug effects, though the amount consumed is not the key factor.

Interestingly, in chow-free mERC, vehicle mice showed robust ITI response ramping (Fig. 3E, left), reduced by haloperidol but largely unchanged by GBR-12909. Neither drug changed response latency (Fig. 3E, right), suggesting that chow availability interacts with response latency and thus may serve as a motivational counterpoint to anticipatory touchscreen responses. Overall, our data indicate that long ITI, increased effort, and the presence of a choice (chow vs. reward) are all required to confer full dopaminergic sensitivity in mERC.

### Neurochemical impact of tetrabenazine and GBR-12909 in rat dorsal striatum and NAc

**Fig. 4:**
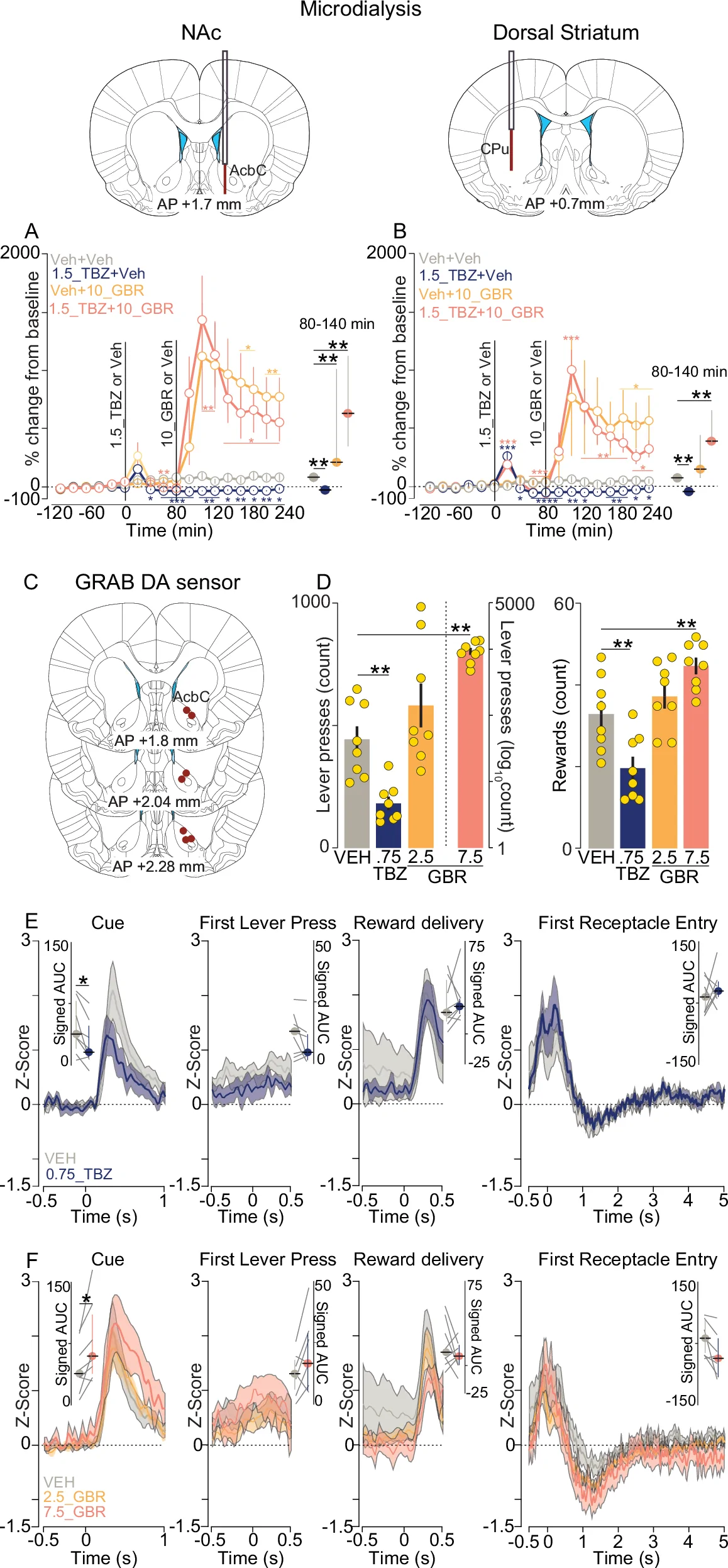
Effects of tetrabenazine and GBR-12909 on rat dorsal striatal and nucleus accumbens dopamine. **A**, **B** Reconstructed microdialysis probe locations (top) and effects of tetrabenazine (*n* = 10), GBR-12909 (*n* = 10), their combination (*n* = 10), or vehicle (*n* = 9; mg/kg) on normalized extracellular dopamine in the **A** nucleus accumbens (NAc) and **B** dorsal striatum (dSTR). Right panels show summed mean values 80–140 min after initial treatment. **C** Optic-fiber placements in the rat brain. **D** Lever presses in mERC-trained rats expressing GRAB_DA2m and implanted with optic fibers (*n* = 8) after vehicle, tetrabenazine, or GBR-12909. NAc dopamine signals (GRAB_DA2m) aligned to cue onset, first lever press, reward delivery, and first receptacle entry after reward; treatment effects of vehicle or tetrabenazine (**E**) and two doses of GBR-12909 (**F**). Insets show median signed AUC (IQR) with individual values in gray. Statistics in Supplementary Table S10.

To evaluate how tetrabenazine and GBR-12909 affect dopamine transmission across timescales, we performed in vivo microdialysis and fiber photometry in rats. Microdialysis showed that 1.5 mg/kg tetrabenazine produced a sustained reduction in extracellular dopamine in both the nucleus accumbens and dorsal striatum (Fig. 4A, B; blue trace), while 10 mg/kg GBR-12909 produced a prolonged increase (yellow trace). Co-administration fully reversed tetrabenazine’s depleting effect, restoring dopamine to GBR-12909 levels (red trace). These neurochemical effects align with the behavioral data: tetrabenazine reduces lever pressing in both sERC and mERC. However, GBR-12909 alone enhances responding only in mERC, suggesting that mERC behavior is sensitive to experimentally induced changes in extracellular dopamine levels.

We then used fiber photometry with the GRAB_DA2m sensor [43] to measure phasic dopamine signals during mERC. In a crossover design, tethered rats showed comparable behavior (Fig. 4D) as non-tethered ones (Fig. 1F). Time-locked analyses revealed that tetrabenazine reduced cue-evoked dopamine signals, while GBR-12909 increased them (Fig. 4E, F, left panels). Together with the microdialysis data, these results support the involvement of dopaminergic signaling across timescales. However, although these complementary measures assess dopamine dynamics at different temporal resolutions, they do not directly test how tonic and phasic signaling independently contribute to mERC behavior, nor whether task design alters tonic dopamine during task performance.

### Limited dopamine depletion in mice by tetrabenazine reflects weak behavioral effects

Unlike the consistent effects in rats, reports on the behavioral impact of tetrabenazine in mice have been mixed, with some studies finding strong effects and others showing little to none [19, 21, 29, 60–62]. To clarify this discrepancy, we investigated three doses of tetrabenazine in rat and mouse using MALDI-MSI—which enables semi-quantitative spatially resolved measurement of neurochemical distributions in ex vivo brain sections.

**Fig. 5:**
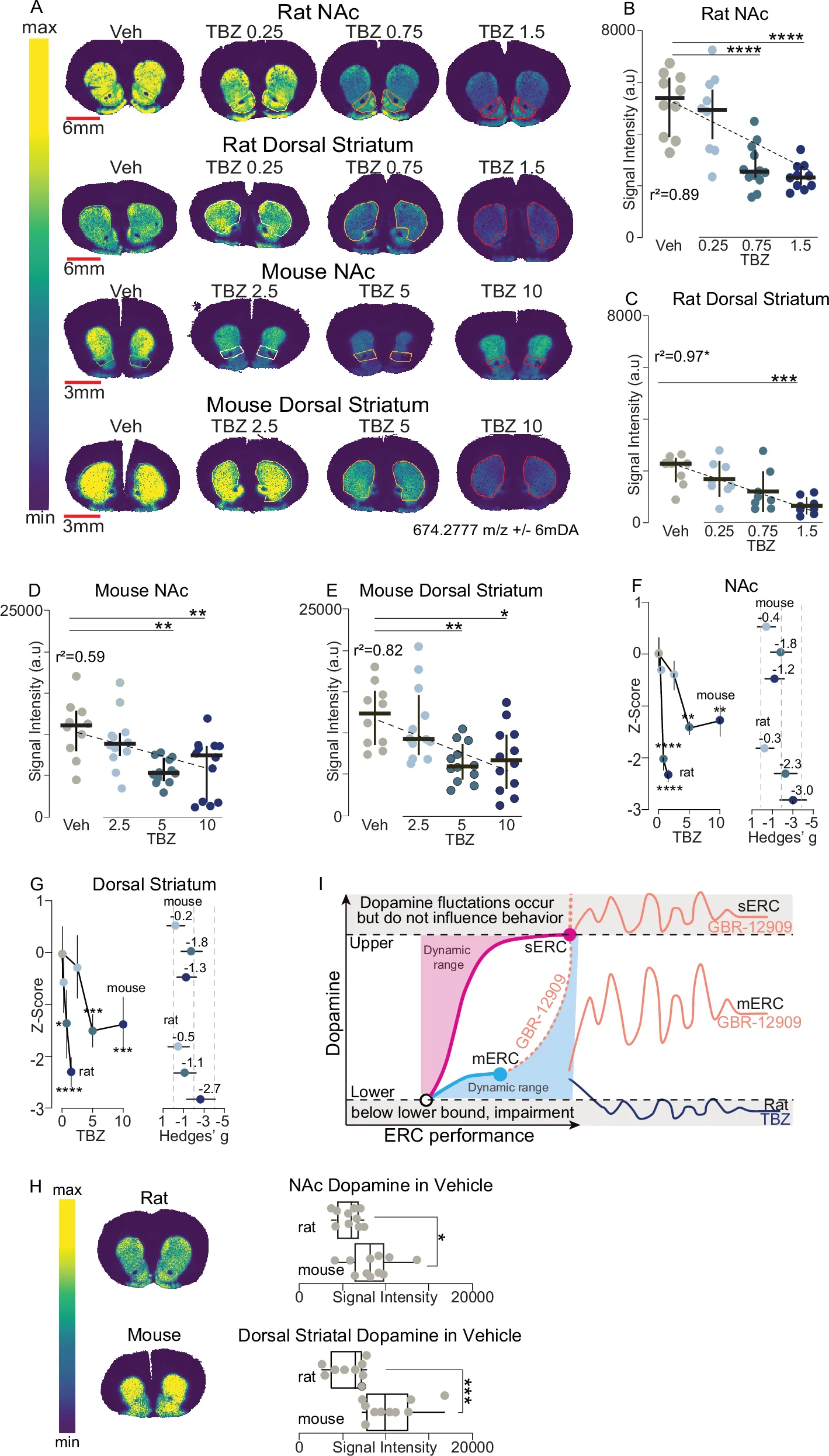
Species-specific DA depletion by tetrabenazine shown by MALDI-MSI. **A** Representative MALDI-MSI images of dopamine in coronal sections of rat NAc (*n* = 5 vehicle, 6/group) and dorsal striatum (*n* = 4/group), and mouse NAc and dorsal striatum (*n* = 5 vehicle, 6/group) after vehicle or tetrabenazine (mg/kg). **B**–**E** Median dopamine signal intensity (IQR) across tetrabenazine dose–response with overlaid linear regression; points represent individual subjects including technical replicates. Z-scored tetrabenazine effects on dopamine in rat and mouse NAc (**F**) and dorsal striatum (**G**), with corresponding Hedges’ g effect sizes. **H** Representative dopamine levels in rat and mouse NAc and dorsal striatum after vehicle (left) and quantification of corrected dopamine intensities (right). **I** Schematic summary proposing that dopamine modulates behavior within a bounded dynamic range, with impairment below a lower limit and saturation above an upper limit. Statistics in Supplementary Table S11.

In rat, we observed a clear dose-dependent dopamine depletion in nucleus accumbens and dorsal striatum, with near-complete depletion at 1.5 mg/kg tetrabenazine (Fig. 5A, top panels). This result aligns with the dose–response observed in mERC (Fig. 1F) and sERC (Fig. 1C). In mice, doses ~10-fold higher than in rats (Fig. 2B) produced clear dorsal striatal depletion at 10 mg/kg, while dopamine in the nucleus accumbens remained substantial and highly variable (Fig. 5A).

Plotting signal intensity by dose and brain region (Fig. 5B–E) showed a linear dose–response in rats, whereas mice displayed a less linear pattern that plateaued at higher doses with weaker maximum depletion (Fig. 5B–E). Z-scores and Hedges’ *g* confirmed significantly greater depletion in rats than in mice (Fig. 5F, G). Notably, increasing the mouse dose above 5 mg/kg did not further deplete dopamine on average and produced substantial inter-individual variability (Fig. 5D).

These results indicate that tetrabenazine is a relatively weak dopamine‑depleting agent in mice under the doses tested, consistent with its limited behavioral efficacy in mouse mERC. Finally, we directly compared baseline dopamine levels in NAc and dorsal striatum across species (Fig. 5H). Compared to rats, vehicle‑treated mice showed ~1.35‑fold higher dopamine levels in NAc and ~1.75‑fold higher dorsal striatum. Combined with the comparatively modest depletion in mice (<2‑fold at high dose vs. >3‑fold in rats), this supports a straightforward explanation for reduced behavioral effects in mice: higher baseline dopamine and weaker depletion likely prevent tetrabenazine from lowering dopamine below a threshold required to consistently impair mouse ERC performance.

## Discussion

Our findings establish the modified Effort-Related Choice task (mERC) as a robust cross‑species task for measuring motivated behavior that is highly sensitive to both dopaminergic enhancement and impairment. In contrast to standard ERC (Figs. 1A and 2A), which showed limited sensitivity to dopamine-enhancing drugs unless paired with pharmacological challenges (Fig. 1B–E), mERC—by incorporating higher effort demands, fixed long ITIs, and explicit cueing—revealed clear, bidirectional, dose‑dependent drug effects within a single task. Dopamine-enhancing compounds (GBR-12909, amphetamine, and modafinil) reliably increased effortful responding, whereas dopamine-impairing manipulations (tetrabenazine, haloperidol, and the A2A agonist CGS-21680) reduced performance (Figs. 1F–L and 2F–G). These behavioral effects were accompanied by systematic changes in response vigor, including anticipatory behaviors during the ITI and alterations in response latency (Figs. 1H–J and 2H–J).

In mice, freely available chow was essential for revealing pharmacological sensitivity in mERC (Fig. 3D–F), underscoring the importance of task and choice structure for engaging dopaminergic control. As in sERC, rat mERC showed an inverse relationship between effort expenditure and chow intake, whereas chow consumption in mice remained largely unchanged across conditions (Supplementary Fig. S1; Supplementary Tables S5–S10). Together with the microdialysis and photometry results, these findings support a role for both tonic and phasic dopamine signaling during task performance (Fig. 4), providing a framework for linking behavioral and neurochemical measures of effortful action. However, despite this convergent evidence, the proposed model remains untested (see limitations below).

Unlike tasks that adapt sERC using fixed, random, variable, or progressive ratio schedules [63] to dissociate motivational impairment from enhancement, mERC enables direct, within-task comparisons of dose-dependent drug effects. Progressive ratio procedures, while valuable, pose challenges for pharmacological studies due to substantial inter- and intra-subject variability in breakpoint, species differences, and sensitivity to pharmacokinetics. Although pro-motivational effects of dopamine manipulations have been demonstrated using alternative paradigms—combined with genetic manipulations [63, 64] or pharmacology [65]—these tasks typically lack the temporal precision, stability, and within-task consistency provided by mERC. By contrast, mERC offers a scalable and controlled platform for pharmacological testing that preserves sensitivity across species.

Dopamine dependence in rodent behavioral tasks scales with effort demands (for review, see ref. [66]). Low-effort tasks show little sensitivity to dopamine modulation, whereas moderate-to-high effort conditions more robustly engage the dopamine system—particularly within the nucleus accumbens, where dopamine signals reward value [67], effort cost [68] and action initiation. High-effort rewards elicit greater dopamine release [68, 69] and are perceived as more valuable [70], yet several findings challenge a strict cost-benefit interpretation of dopamine’s role. Weak effort encoding in phasic dopamine signals [71] and the absence of effort bias following dopamine neuron lesions [72] instead support a view of dopamine as an incentive signal that promotes action initiation rather than encoding effort costs per se (for review see ref. [73]. Discrepancies across studies may reflect methodological differences, species differences, and the timing of dopamine measurements—specifically, whether signals are captured after effort expenditure (sunk cost) [68] or during anticipation of future effort [71, 74, 75]. Our data supports a context-dependent role for dopamine: high effort alone revealed sensitivity to dopamine impairment (Fig. 2B), whereas acute dopamine enhancement increased reward-seeking and anticipatory behavior only when combined with prolonged ITIs (Fig. 2F, H).

A key optimization in mERC is the inclusion of a long-fixed ITI. Under the flexible approach hypothesis [76], extended ITIs promote trial-by-trial integration of reward paths and enhance dopamine dependency. Our findings support this framework while also highlighting the necessity of effort demands. High effort in the absence of an ITI did not permit pro-motivational effects of GBR-12909 (Fig. 2B), likely because animals self-imposed short, variable pauses that limited anticipation behaviors. Conversely, low effort paired with a long ITI produced weak, unstructured anticipatory responses that did not translate to increased reward (Fig. 2C, D). Only the combination of high effort and a long ITI enabled dopamine-enhancing drugs to elicit robust anticipatory behaviors that shortened response latencies and increased reward acquisition by reducing opportunity cost (Figs. 1I, J and 2H, J). Importantly, drug-induced changes in ramping ITI responses (Fig. 1H, I) indicate alterations in when effort is allocated, rather than a general increase in effort expenditure.

This ramp-like response profile resembles interval-timing behavior in peak interval procedures [77–79], which depends on striatal dopamine tone: elevated tonic dopamine accelerates the internal clock regulating reward seeking, while low levels slow it down [80, 81] (for review and commentary see refs. [82–84]). The effects of dopamine modulators on response latency and anticipatory pressing in mERC (Figs. 1H–J and 2H–J) align with interval timing impairments observed in Parkinson’s disease [85–87] and the beneficial effects of dopaminergic treatments [88]. These drug-induced enhancements likely reflect increased behavioral vigor [16, 89] but also suggest a shift in effort-value decision driven by internal timing cues [10, 83], producing shorter latencies between action and intention. Such mechanisms may be particularly relevant to clinical conditions characterized by anticipatory anhedonia [90–92] and effort-related inaction [2, 32, 33, 39] where reducing action initiation latency could yield therapeutic benefit.

Neurochemical measures further support this interpretation and are intended not as standalone discoveries, but as constraints that delineate when and where sensitivity to dopaminergic modulation can emerge (Fig.4). Systemic pharmacological manipulations produced predictable and bidirectional changes in extracellular dopamine levels and cue-evoked signals in the nucleus accumbens and dorsal striatum, closely paralleling behavioral effects in mERC.

Tetrabenazine reduced extracellular dopamine and attenuated cue-locked dopamine transients [18], whereas GBR-12909 increased both measures and reversed tetrabenazine’s effects when co-administered. Although interpretation of GRAB_DA signal under pharmacological manipulation is constrained by baseline normalization and drug-induced shifts in tonic dopamine (see ref. [93]), the observed changes demonstrate that mERC engages fast phasic dopaminergic signaling. Whether these changes reflect altered cue salience, proximity to the lever at cue onset, or expectations regarding future effort—and how these mechanisms differ across striatal subregions—remains an important question for future studies, including direct causal tests using optogenetic manipulations of phasic dopamine during task performance.

In mice, tetrabenazine produced weak and variable behavioral effects (Fig. 2A, B, F), consistent with higher baseline dopamine levels (Fig. 5H) and incomplete depletion (Fig. 5D, E). MALDI-MSI revealed substantially higher baseline dopamine in mouse NAc and dorsal striatum compared to rats alongside a plateau in dopamine depletion at higher doses with marked inter-individual variability. Depletion in mouse NAc was particularly inconsistent, suggesting that regional sensitivity, species-specific pharmacokinetics, compensatory mechanisms, or non-VMAT2 processes may contribute to regulation of dopamine tone. Importantly, residual dopamine levels in mice likely remain above a critical lower threshold required to consistently impair ERC performance, providing a parsimonious explanation for the limited behavioral efficacy of tetrabenazine in this species.

Our neurochemical and pharmacological data support the concept that dopamine operates within a bounded dynamic range for behavioral modulation (see theoretical model in Fig. 5I), like patterns seen in cognitive performance [94, 95]. In mERC, performance is impaired only when dopamine falls below a critical lower limit, whereas the lack of GBR-12909 effects in sERC suggests that baseline dopamine in this task context already meets or exceeds an upper limit, beyond which additional enhancement offers no benefit and may even impair performance (e.g., amphetamine in Fig. 1A and other dopaminergic agents [94, 96–99]). Within this window, tonic dopamine may set proximity to behaviorally relevant thresholds, while phasic transients superimposed on tonic levels drive rapid state transitions and modulate reward seeking. Outside this range, dopamine fluctuations may still occur but become decoupled from behavior.

Importantly, we did not directly measure tonic dopamine under different task conditions, and thus direct confirmation of our proposed model of task-dependent tonic dopamine levels (Fig. 5I) will require quantitative approaches capable of measuring tonic dopamine during behavior. Therefore, any interpretation linking task structure to tonic dopamine levels remains hypothetical.

Adopting an RDoC-inspired [100] approach, this work highlights mERC as a valuable translational tool for isolating symptom‑relevant biological mechanisms and clarifies how task architecture and dopaminergic state interact to shape motivated behavior. Beyond its utility for drug screening, mERC provides a platform for mechanistic dissection of effortful decision-making: combining the task with circuit‑specific manipulations, cell‑type‑specific recordings, or targeted interventions during the ITI could further elucidate how anticipatory processes drive effort allocation. Given recent reported sex differences in tetrabenazine’s behavioral effects [101–103], future studies should extend mERC to female rodents. Application of mERC in disease-relevant or stress-based models represents another critical avenue for future investigation.

In summary, mERC offers a powerful and scalable platform for evaluating effort‑based decision-making and motivated behavior across rodent species. By integrating high effort demands, extended ITIs, and explicit choice, mERC enables sensitive detection of both impairing and enhancing drug effects within a single task. Our findings underscore the state‑dependence of dopamine, the importance of task structure, and species‑specific pharmacodynamics in shaping motivated behavior, providing a valuable framework for advancing therapeutic strategies targeting motivational deficits in neuropsychiatric and neurological disorders.

## Supplementary information

Supplemental Materials

## Data Availability

Data supporting the findings of this study are available from the corresponding author upon request.
