# Supplementary material for: Effort, timing and choice: a refined rodent effort choice task with excellent sensitivity to dopamine modulators
Source: Neuropsychopharmacology. 2026 Jun 10;51(11):2227–37. doi: 10.1038/s41386-026-02453-8 (PMC13597516; doi:10.1038/s41386-026-02453-8)
Supplement: Supplementary file 1 [file 41386_2026_2453_MOESM1_ESM.pdf]

SUPPLEMENTAL MATERIAL

Animals

Supplemental Table S1. Animal usage

Species	Strain	Sex	N	Weight / age	Supplier	Experiment	Figures
Rat	SD	M	14	230-250g	Janvier Labs, FR	Behavior	1A, 1B
Rat	SD	M	14	230-250g	Janvier Labs, FR	Behavior	1C
Rat	SD	M	13	230-250g	Janvier Labs, FR	Behavior	1D, 1E
Rat	SD	M	14	230-250g	Janvier Labs, FR	Behavior	1F
Rat	SD	M	13	230-250g	Janvier Labs, FR	Behavior	1G, 3B, 3C
Rat	SD	M	12	230-250g	Janvier Labs, FR	Behavior	1J
Rat	SD	M	13	230-250g	Janvier Labs, FR	Behavior	1K
Rat	SD	M	15	230-250g	Janvier Labs, FR	Behavior	3A
Rat	SD	M	40	300g	Janvier Labs, FR	Microdialysis	4A, 4B
Rat	Wistar	M	11	250-270g	Janvier Labs, FR	Fiber photometry	4C-4F
Rat	SD	M	39	360g	Janvier Labs, FR	MALDI-MSI	5
Mouse	C57BL/6JRj	M	16	7-9 w	Janvier Labs, FR	Behavior	2A-2G, 3E, 3F
Mouse	ChAT-IRES-Cre*	M	17	7-9 w	JAX, USA	Behavior	2H, 2I
Mouse	C57BL/6JRj	M	23	8-9 w	Janvier Labs, FR	MALDI-MSI	5

*JAX stock 006410; SD: Sprague Dawley

<u>Food Restriction in behavioral studies</u>

After acclimation to the animal facility, rats and mice undergoing behavioral procedures were weighed for 3 days to establish an average baseline body weight for food restriction. Starting on the first day of restriction, animals were weighed daily for two weeks, followed by weekly (for rats) or daily (except weekends, for mice) weigh-ins to ensure well-being and compliance with weight criteria.

Rats received 15–18g of standard laboratory chow per day, depending on their training stage (see Table 1). Body weights were monitored throughout the study and compared to three reference cohorts from historical data fed (a) ad libitum, (b) 18g/day, or (c) 15g/day. Weight

remained ~90%, with small gains (if any) observed during the experimental period. Mice were fed 2.5–3.5g of chow per day, adjusted daily to maintain ~90% of their initial body weight

General Behavioral procedures

All rat behavioral experiments (Figs. 1 and 3) took place in 36 standard rat operant conditioning chambers (Med Associates Inc, Fairfax, VT, USA) which were operated by custom scripts written in Med-State Notation. Rewards for lever pressing were 50% sucrose pellets (5TUL - Purified Rodent Tablet, Purina Test Diet, St Louis, MI, USA). For mouse (Figs. 2 and 3), 16 Bussey-Saksida touchscreen systems purchased from Campden instruments (80614-0-052, Loughborough, United Kingdom) were used for behavior and operated by custom scripts written in ABET II. In the mouse touchscreen tasks, 10% sucrose solution (60 µl droplet delivered into the reward receptacle) was used as reward. The touchscreen was covered with a custom-made black Perspex mask with 1 window (4x4 cm), either flush to the grided floor (lower target) or raised 5.5 cm (raised target, as indicated below on table S2).

Free chow during sERC and mERC

During the food choice phase, animals had access to 15g of standard laboratory chow (rat) or 2g (one piece) of chow (mouse) located on a small petri dish (plate) secured to the grid floor within the operant chamber. The chow used in this study was 1328 FORTI-Haltungsdiät-ratten/mäuse-fortified (Altromin Spezialfutter GmbH & Co. KG, Germany). At the end of each session, leftover food was collected and weighed to calculate individual food consumption. Leftover food referred to chow remaining on the plate or grid within the chamber, while wasted food was chow collected from the waste tray beneath the grid. See Supplemental Figure 1 for summary of chow consumed during all experiments and Supplemental Tables S5-10 for detailed statistics.

Supplemental Figure 1. Chow consumption during all behavioral experiments

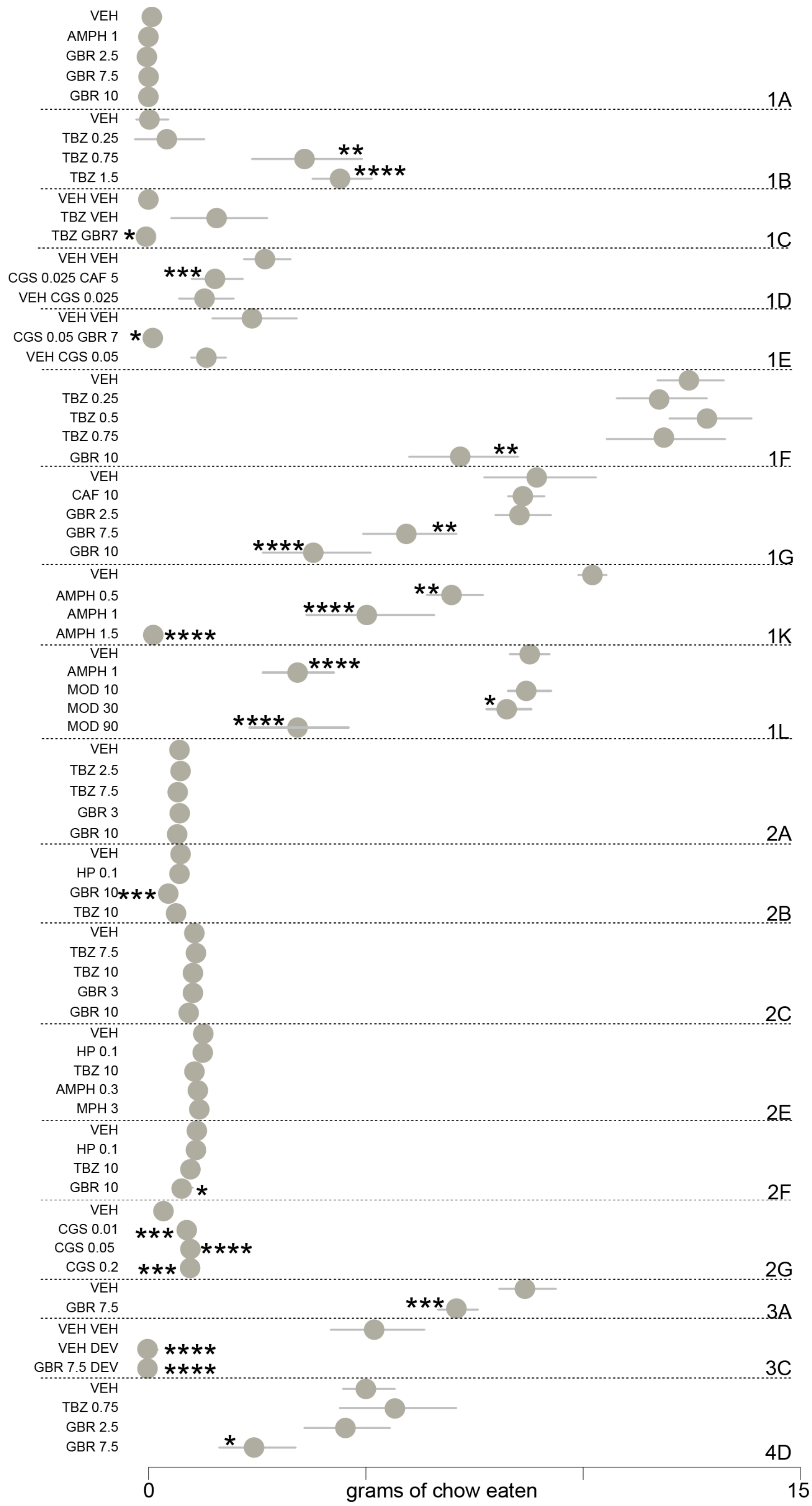

Fig. S1. Chow consumption during sERC or mERC test sessions. Average chow consumption (g) per group for all behavioral experiments with food choice (chow vs reward). Rats: 1A-1K, 3A and 4D. Mice: 2A-2H and 3C. * p < 0.05; ** p< 0.01; *** p < 0.001; **** p < 0.0001. For detailed statistics refer to supplemental tables S5-S10.

Supplemental table S2. Rat Effort Related Choice (ERC) task – training protocol

Stage	Description	Criteria / days of training
**Receptacle training**	Reward pellet is delivered every 30s; reward collection required for next reward (receptacle light signals reward availability). 60 min session	200 rewards; 2-3 days
**FR1**	Lever always accessible; FR1 schedule; receptacle light signals reward which must be collected for lever to become active again; 60 min session	200 rewards; 2-3 days
**FR5**	FR5; 30 min session, 5 days per week	>1000 lever presses; 2-3 days
**FR5/choice**	FR5 + 15g of chow placed in the operant chamber (free access); 30 min, 5 days per week	Until performance is stable. Leftover chow put in home-cage
**Modified Effort Related Choice task (following steps 1-3, skip 4 above)**		
**FR8/choice**	FR8 + 15g of chow in the operant chamber (free access); 30 min, 5 days a week	~1500 lever presses; 3-4 days
**FR8/choice + 30s ITI**	Active lever is signaled by a light cue located above the lever (on = active, off = inactive). Light cue onset 30s after reward on the previous trial is collected (off during ITI).15g chow/rat available in the operant chamber. 30 min, 5 days a week	~1 week to achieve stability of lever presses. Daily food ration may need to be raised from 15 to 18g for weight maintenance

Supplemental table S3. Mouse Effort Related Choice (ERC) task – training protocol

Stage	Description	Criteria / days of training
**Receptacle training**	Reward every 5s, if reward retrieval (receptacle light signals reward availability); 30 min session	>30 rewards; ~ 2 days
**FR1 lower target**	Must touch square located~ at floor level; 60 min session	>30 rewards for 2 consecutive days; ~ 5 days
**FR1 raised target**	Square is raised 5.5 cm from the bottom of the screen; 60 min session	>40 rewards for 2 consecutive days; ~ 5 days
**FR1 choice**	2g of standard chow on a plate in the chamber; 30 min session, 5 days per week	>30 rewards for 2 consecutive days; 5-10 days
**FR1 choice +ITI**	Add a 40s intertrial interval (ITI); 45 min session, 5 days per week	>30 rewards for 2 consecutive days; 5-10 days
**Modified Effort Related Choice task (following steps 1-3, skip 4-5 above)**		
**FR5 raised target**	Same as in **FR1 raised target**, but 5 touches required for reward; 45 min session	> 40 rewards; 2-3 days
**FR8 raised target**	Same as above, but 8 touches required for reward; 45 min session	> 40 rewards for 2 consecutive days; ~ 5 days
**FR8 raised target +choice**	Same as above + plate with one piece (2g) of chow on the floor of the chamber; 45 min session	> 30 rewards for 2 consecutive days; ~ 5 days
**FR8 raised target choice +ITI**	Same as above, but with a 30s intertrial interval (ITI) between reward retrieval and cue onset; 45 min session	> 20 rewards for 2 consecutive days; ~ 5 days

Supplemental Figure 2. mERC rat and mouse training

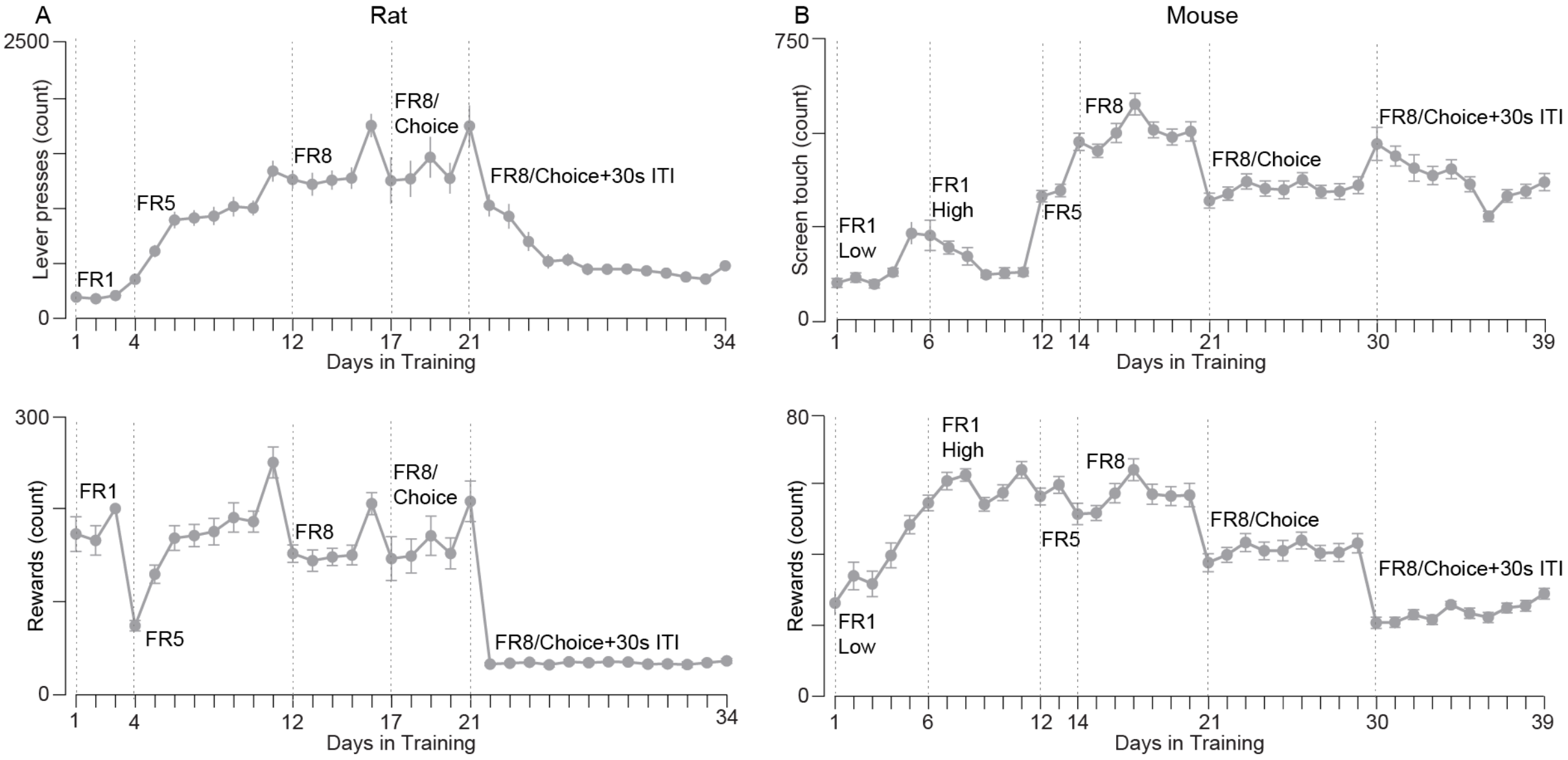

Fig. S2. Training curves for rat and mouse mERC. Average within cohort lever presses / screen touches (top) and rewards (bottom) for rat (A) and mouse mERC (B) for all training stages outlined in Supplemental Tables S2-S3.

Supplemental table S4. Details of the drugs used in the study

Drug	Species	Route	Dose (mg/kg)	Pre-Treat	Formulation	Volume (mL/kg)	PH	Figures
Amphetamine	Rat	i.p.	0.5, 1, 1.5	30 min	0.9% saline	1	7	1A, 1J
	Mouse	i.p.	0.3	15 min	0.9% saline	10	7	2E
Caffeine	Rat	i.p.	10	20 min	0.9% saline	1	7	1D, 1G, 1K
CGS-21680	Rat	i.p.	0.05,0.1,0.2	15 min	2%DMSO+ 20% HBCD in 0.9% saline	1	7	1D, 1E
	Mouse	i.p.	0.01, 0.05, 0.2	15 min	2% DMSO in 0.9% saline	10	7	2H
GBR-12909	Rat	i.p.	2.5, 7.5, 10	30 min	20% HPBCD in H_2O	1	7	1C, 1E-1G, 3A-3C, 4
	Mouse	i.p.	3, 10	30 min	20% HPBCD in H_2O	10	7	2A-2G, 3D, 3E
Haloperidol	Mouse	s.c.	0.1	60 min	20% HPBCD in H_2O	10	5	2B, 2E-2G, 3D, 3E
Modafinil	Rat	i.p.	10, 30, 90	30 min	0.5% Na-CMC + 0.015%Tween80 in 0.9% saline	5	7	1K
Tetrabenazine	Rat	i.p.	0.25, 0.5, 0.75, 1.5	90 min	20% HPBCD in 0.9% saline	1	7	1B, 1C, 1F, 5
	Rat	i.p.	1.5	90 min	10% DMSO in 0.9% saline	1.5	3.5	4A-4B
	Mouse	i.p.	2.5, 7.5, 10	90 min	20% HPBCD in 0.9% saline	10	7	2A-2G, 5

***HPBCD:** 2-Hydroxypropyl-β-cyclodextrin

Additional Details: All drugs, except CGS-21680 (Tocris Bioscience, Bristol, United Kingdom) and Tetrabenazine (Sigma-Aldrich, Steinheim am Albuch, Germany) were supplied by Boehringer Ingelheim (Biberach an der Riss, Germany).

Microdialysis

Stereotaxic surgeries for implantation of microdialysis guide cannulae and sampling procedure were performed as previously described [1]. Briefly, rats were anesthetized and head-fixed to a stereotaxic frame. Holes were drilled in their skulls to implant intracerebral guide cannulae (*MAB 4.9.IC, dSTR, and MAB 4.15.IC, NAc, Microbiotech, Sweden) targeting: dorsal striatum (AP +0.7 mm, ML +3.0 mm, DV -4.5 mm from bregma) and the nucleus accumbens (AP +1.7 mm, ML -1.5 mm, DV -6.0 mm from bregma). Post-surgery, rats received daily Meloxicam (Boehringer Ingelheim, Biberach an der Riss, Germany) for 5 days and were monitored for well-being. After a week of recovery, microdialysis probes (MAB 4.15.2 Cu and MAB 4.9.2 Cu: 2 mm membrane length, 6 kDa cut-off) were inserted into the cannulae of awake rats which were then perfused with standard aCSF solution (147 mM NaCl, 2.7 mM KCl, 1.2 mM $CaCl_2$, 0.85 mM $MgCl_2$ and 1 mM Na_2HPO_4, pH 7.2). Dialysate samples were collected every 20 minutes, starting 2 hours after probe insertion. For experiments, rats were placed in individual clear plexiglass chambers (30cm x 40cm) and allowed to freely move for the duration of the sampling period. Rats were dosed with tetrabenazine (1.5 mg/kg i.p.; 1 ml/kg) or vehicle (10% DMSO in saline, pH 3.5), followed by GBR-12909 (10 mg/kg i.p.; 1,5 ml/kg) or vehicle (20% HP-ß-CD) 80 min after. Sampling continued every 20 min for an additional 160 min. Probe localization was verified histologically (The Rat Brain in Stereotaxic Coordinates, Paxinos and Watson, Academic Press, 2013), and samples were frozen and stored at -80°C for later analysis of dopamine concentrations using LC-MS/MS. The setup consisted of an Agilent 1290 Infinity II Multisampler and High-Speed Pump (Waldbronn, Germany), coupled to an ABSciex 7500 Triple Quadrupole mass spectrometer (Darmstadt, Germany). Quantification was performed using calibration curves generated from reference standards prepared in artificial cerebrospinal fluid (aCSF). Isotopically labeled internal standards were used to ensure accuracy and precision. Instrument control, data acquisition, and processing were carried out using SciexOS software version 3.4.5.

The analytical method was based on and modified from Wong et al. [2]. Chromatographic separation was achieved using an ACQUITY Premier UPLC HSS T3 column (2.1 × 100 mm, 1.8 µm). A 10 µL sample volume was injected at a flow rate of 0.5 mL/min under a gradient elution program, with the column maintained at 40°C. Mobile phase A consisted of 10 mM ammonium formate with 0.15% formic acid in water, while mobile phase B contained 10 mM ammonium formate with 0.15% formic acid in 90% acetonitrile. The mass spectrometer operated in positive electrospray ionization (ESI) mode, with optimized parameters for each analyte.

LC-MS Sample Preparation

A 20 µL aliquot of dialysate was derivatized using a modified protocol. Sequential additions were made with vortex mixing after each step: 5 µL of internal standard mixture, 15 µL of 100 mM sodium carbonate, 10 µL of 2% benzoyl chloride in acetonitrile, and 25 µL of 5 mM citric acid containing 0.1% formic acid.

Analysis

Neurotransmitter levels were normalized to the 60-minute pre-drug baseline, corrected to zero and expressed as percent change.

Fiber photometry

Hardware

The fiber photometry acquisition system consisted of two RZ10x processors (Tucker-Davis Technologies, FL, USA), each equipped with 405 nm violet and 465 nm blue LEDs to measure dopamine-independent and dopamine-dependent GRAB_DA2m(DA4.4) fluorescence, respectively. The 405 nm LED was modulated at 210 Hz, while the 465 nm LED was modulated at 330 Hz. LEDs were connected to fluorescence mini cubes (Doric Lenses, Canada) via drive cables (LUX cable kit, Tucker-Davis Technologies, FL, USA), which were further linked to pigtailed fiber-optic rotary joints mounted on top of the operant chambers. Patch cords (400 µm core, Doric Lenses, Canada) extending from the optical swivels were

connected to the rats inside the operant boxes. Each patch cord was attached to the implanted fiber optic cannula on the rat's head using a metal ferrule or zirconia sleeve connector (Doric Lenses, Canada).

Each RZ10x processor could record from two subjects simultaneously, allowing data collection from up to four subjects at a time. The Synapse software suite (Tucker-Davis Technologies, FL, USA) controlled the processors, recorded data, and synchronized behavioral and photometric signals via TTL pulses sent through a passive TTL connection panel (Med Associates, Vermont, USA) integrated with the operant boxes. To minimize autofluorescence, fiber optic patch cords were photobleached for at least one hour on each experiment day using the bleach setting in the Synapse software. Voltage output settings were customized for each subject based on initial signal screening and remained unchanged unless a significant signal decrease was detected, in which case the voltage was adjusted.

AAV injection and optic fiber implants

Stereotaxic surgeries for viral vector injections and fiber optic implantation were performed similarly to as previously described [3]. Briefly, 800 nL of AAV1/2-hSyn-GRAB_DA2m(DA4.4) (1.10E+12 VG/mL) was unilaterally injected into the nucleus accumbens of rats using a 5 µL Hamilton microsyringe at a rate of 200 nL/min. Injection coordinates were measured from the skull at bregma (AP: +1.2 mm, ML: ±2.0 mm, alternated for each subject; DV: -7.2 mm from dura mater). Following the injection, the site was sealed with bone wax, and rats were sutured, weighed, and monitored in a warm cage until full recovery. Post-surgery, rats received daily Meloxicam (Boehringer Ingelheim, Biberach an der Riss, Germany) for 5 days and monitored daily for well-being.

After one week of recovery, the animals were food restricted and trained on the modified effort choice task (mERC) as described in Supplemental Table 2. Five weeks post-first surgery, rats underwent optic fiber implantation surgery (10mm long and 400µm thick with a borosilicate core, Doric Lenses). Cannulae were chronically implanted above the virus injection site in the

nucleus accumbens (DV: -7.0 mm from dura mater). Four bone screws were fitted to the skull, and the optic fibers and screws were secured with dental cement.

Data acquisition and analysis

After recovery from fiber optic implantation, the animals were re-trained on mERC for ~1 week to acclimate to the task when attached to the fiber optic rotary joint by a patch cable. Pharmacology was done in an identical manner as described in the main methods section "Drugs". Briefly, animals received vehicle, 0.75mg/kg tetrabenazine, 2.5 or 10mg/kg GBR-12909 in a crossover design where drugs are administered Tuesdays and Fridays.

A total of 11 rats were used in the study; 3 were excluded due to either loss of implant or poor signal quality. For preprocessing, GRAB-DA sensor signals were down-sampled to 40 Hz. The autofluorescence channel was subtracted from the signal channel, and the resulting trace was detrended using a non-linear decay model fitted with the poly function in R.

To align neural signals with behavior, TTL flags from Med Associates were used to precisely time-lock the corrected signals to key behavioral events (cue onset, first lever press, reward delivery, and first receptacle entry). Using a 4-second baseline preceding each trial, trial-by-trial z-scores were calculated for a 2-second window following each event onset. These z-scores were first averaged within each animal per session, and then across animals within each condition to generate peri-event time histograms (PETHs). Signals across drug conditions were compared using R (pROC package) to calculate signed area under the curve (AUC) values, enabling detection of both positive and negative effects. AUCs were computed for each animal and drug condition (see Table S10 for statistical details). Finally, we plotted the mean signal transitions and AUCs for each event type across trial types.

Histology

After completion of the experiments, rats were transcardially perfused with PBS, then 4% paraformaldehyde (PFA). Next, brains were postfixed for 48h at 4°C and transferred to a 30% sucrose solution (in PBS) for cryoprotection at 4°C until slicing.

Coronal brain slices (100 µm) were made with a freezing microtome (-27°C, HM 450, Thermo-Fisher Scientific, Waltham, MA, U.S.A). Slices were permeabilized for 2h at room temperature (RT) in a solution containing 1% BSA, 0.3% Triton, and 10% Normal Goat Serum diluted in 1x PBS. They were then incubated overnight at 4°C with an anti-GFP rabbit-polyclonal antibody (#A6455, Thermo Fisher Scientific, Waltham, MA, USA) at 1:200 dilution in 1% BSA, 0.3% Triton, and 1% Normal Goat Serum diluted in 1x PBS. The following day, slices were washed for 45 minutes in 1x PBS/0.1% Triton-X 100 and incubated for 2h in the dark with an anti-rabbit goat Alexa Fluor® 488 secondary antibody (#A11070, Invitrogen, Waltham, MA, USA) at 1:1000 dilution in the same buffer as the primary antibody.

Finally, slices were washed for 60 minutes in 1x PBS/0.1% Triton-X 100, mounted onto glass-bottom 24-well Sensoplates (#662892, Greiner, Kremsmuenster, Austria), air-dried, and covered with 500 µl DAPI-containing Fluoroshield® (#F6057, Sigma-Aldrich, Saint Louis, MO, USA). Plates were sealed with black foil stickers and imaged using the High Content Screening System “Opera Phenix” (Perkin Elmer, Norwalk, CT, USA). Fluorescence images were captured at 5x magnification with exposure times of 200 ms (DAPI), 500 ms (native GFP), and 200 ms (anti-GFP staining) at 100% intensity. Viral targeting and optic fiber placement were verified and confirmed for all, except 1 rat, where brain tissue was damaged during slicing.

MALDI-MSI

For a detailed protocol, please refer to [4,5]. In brief, brain slices were spray-coated with FMP-10 ($C_{20}H_{14}FN^{+}$) reactive matrix (10 mg FMP-10 dissolved in 70% ACN) using an automated pneumatic sprayer (TM-Sprayer, HTX imaging). The matrix was applied in 20 passes (flow rate of 80 µL/min, gas pressure of 6 p.s.i, nozzle velocity of 110 cm/min, nozzle temperature of 70°C, track spacing of 2 mm in horizontal passes). All MALDI-MSI measurements were conducted on a timsTOF fleX (Bruker Daltonics, Bremen, Germany) in positive ion mode. Data acquisition parameters were specified in timsControl (v.6.0.2) and flexImaging (v.7.5) (Bruker Daltonics). Laser power was optimized initially and kept constant. The laser operated at a

frequency of 10 kHz using 200 shots per spectra with a raster width of 50 μm. Mass spectra were acquired covering m/z 150-1500. The instrument was externally calibrated with red phosphorus, and tissue sections were analyzed randomly.

After MALDI-MSI, FMP-10 matrix was washed off by immersing the slides in 70% ethanol for 5 minutes, followed by H&E staining. H&E-stained tissue sections digitized on an AxioScan.Z1 slide scanner (Carl Zeiss, Oberkochen, Germany), equipped with a 20x magnification objective. Image visualization and conversion to TIFF format was done using the ZEN 3.1 blue edition software (Carl Zeiss). MSI data were imported in SCiLS Lab (v.2025a) (Bruker Daltonics) and the digital images were co-registered to the mass spectrometric data. Mass spectra were normalized to total ion count. Striatal areas were annotated under visual guidance using the post-acquisition H&E stain. Neurotransmitters were assigned using accurate mass after derivatization process (±6 mDa tolerance window). The maximum peak intensity of the mean spectrum per region was used as a readout. MALDI-MSI images depict the distribution of the most intense ion of the derivatized dopamine ([DA+ 2[FMP-10]-CH3]+: m/z 674.2807). Hot spot removal was performed. To readout peak intensities, the maximum peak intensity of the mean spectrum per region was used.

Rat vs mouse comparison

In Figure 5H, vehicle treated animals (n= 4 rats and 5 mice for dSTR and n= 5 rats and 5 mice for NAc, 2 technical replicates per animal) were analyzed to compare baseline dopamine signal intensities from the dorsal striatum (rat: bregma 0.62 – 0.50 mm; mice: bregma 0.74 – 0.14 mm) and nucleus accumbens (rat: AP 1.80 – 1.60 mm; mice: bregma 1.34 – 1.18 mm). MALDI imaging data from rat and mouse were imported into SCiLS Lab (v.2025a) and subjected to hot spot removal and normalization by total ion count (TIC). Average dopamine signal intensities were extracted of each sample and visualized using boxplots for direct comparison between species.

Supplemental Table 5 (S5): Figures 1A – 1G

Fig	Comparison$_{(mg/kg)}$	Lever press	Reward	Chow consumption	N
**1A**	AMPH 1 vs. Veh	$F_{press\ (1.938,\ 25.20)}$= 12.24, p= 0.0002, p_{adjust} < ****0.0001	$F_{rew\ (1.732,\ 22.52)}$= 10.29, p= 0.0010, p_{adjust} < ****0.0001	$F_{chow\ (1.151,\ 14.96)}$= 2.754, p= 0.1145, p_{adjust} =0.3208	14
**1A**	GBR 2.5 vs. Veh	$F_{press\ (1.938,\ 25.20)}$= 12.24, p= 0.0002, p_{adjust} =0.5043	$F_{rew\ (1.732,\ 22.52)}$= 10.29, p= 0.0010, p_{adjust} =0.3235	$F_{chow\ (1.151,\ 14.96)}$= 2.754, p= 0.1145, p_{adjust} =0.5687	14
**1A**	GBR 7.5 vs. Veh	$F_{press\ (1.938,\ 25.20)}$= 12.24, p= 0.0002, p_{adjust} =0.8775	$F_{rew\ (1.732,\ 22.52)}$= 10.29, p= 0.0010, p_{adjust} =0.9733	$F_{chow\ (1.151,\ 14.96)}$= 2.754, p= 0.1145, p_{adjust} =0.3184	14
**1A**	GBR 10 vs. Veh	$F_{press\ (1.938,\ 25.20)}$= 12.24, p= 0.0002, p_{adjust} =0.7371	$F_{rew\ (1.732,\ 22.52)}$= 10.29, p= 0.0010, p_{adjust} =0.9993	$F_{chow\ (1.151,\ 14.96)}$= 2.754, p= 0.1145, p_{adjust} =0.2301	14
**1B**	TBZ 0.25 vs. Veh	$F_{press\ (2.028,\ 26.36)}$= 77.62, p < 0.0001, p_{adjust} =**0.0074	$F_{rew\ (1.966,\ 25.56)}$= 76.66, p < 0.0001, p_{adjust} =**0.0087	$F_{chow\ (2.021,\ 26.27)}$= 26.46, p < 0.0001, p_{adjust} =0.0855	14
**1B**	TBZ 0.75 vs. Veh	$F_{press\ (2.028,\ 26.36)}$= 77.62, p < 0.0001, p_{adjust} =< ****0.0001	$F_{rew\ (1.966,\ 25.56)}$= 76.66, p < 0.0001, p_{adjust} =< ****0.0001	$F_{chow\ (2.021,\ 26.27)}$= 26.46, p < 0.0001, p_{adjust} =**0.0013	14
**1B**	TBZ 1.5 vs. Veh	$F_{press\ (2.028,\ 26.36)}$= 77.62, p < 0.0001, p_{adjust} =< ****0.0001	$F_{rew\ (1.966,\ 25.56)}$= 76.66, p < 0.0001, p_{adjust} =< ****0.0001	$F_{chow\ (2.021,\ 26.27)}$= 26.46, p < 0.0001, p_{adjust} =< ****0.0001	14
**1C**	TBZ 0.75 + Veh vs. Veh	$F_{press\ (1.708,\ 18.79)}$= 20.35, p < 0.0001, p_{adjust} =< ****0.0001	$F_{rew\ (1.882,\ 24.46)}$= 21.21, p < 0.0001, p_{adjust} =***0.0001	$F_{chow\ (1.355,\ 17.61)}$= 7.356, p= 0.0093, p_{adjust} =0.0691	14
**1C**	TBZ 0.75 + GBR 7 vs. Veh	$F_{press\ (1.708,\ 18.79)}$= 20.35, p < 0.0001, p_{adjust} =0.9959	$F_{rew\ (1.882,\ 24.46)}$= 21.21, p < 0.0001, p_{adjust} =0.7924	$F_{chow\ (1.355,\ 17.61)}$= 7.356, p= 0.0093, p_{adjust} =0.5769	14
**1C**	TBZ 0.75 + GBR 7 vs. TBZ + Veh	$F_{press\ (1.708,\ 18.79)}$= 20.35, p < 0.0001, p_{adjust} =**0.0018	$F_{rew\ (1.882,\ 24.46)}$= 21.21, p < 0.0001, p_{adjust} =***0.0002	$F_{chow\ (1.355,\ 17.61)}$= 7.356, p= 0.0093, p_{adjust} =*0.0156	14
**1D**	CGS 0.025 vs. Veh	$F_{press\ (1.550,\ 18.59)}$= 32.53, p < 0.0001, p_{adjust} =< ****0.0001	$F_{rew\ (1.359,\ 16.31)}$= 30.13, p < 0.0001, p_{adjust} =< ****0.0001	$F_{chow\ (1.571,\ 18.85)}$= 10.15, p= 0.0190, p_{adjust} =0.0525	13
**1D**	CGS 0.025 + CAF 5 vs. Veh	$F_{press\ (1.550,\ 18.59)}$= 32.53, p < 0.0001, p_{adjust} =0.5345	$F_{rew\ (1.359,\ 16.31)}$= 30.13, p < 0.0001, p_{adjust} =0.5613	$F_{chow\ (1.571,\ 18.85)}$= 10.15, p= 0.0190, p_{adjust} =***0.0005	13
**1D**	CGS 0.025 + CAF 5 vs. CGS + Veh	$F_{press\ (1.550,\ 18.59)}$= 32.53, p < 0.0001, p_{adjust} =***0.0004	$F_{rew\ (1.359,\ 16.31)}$= 30.13, p < 0.0001, p_{adjust} =***0.0006	$F_{chow\ (1.571,\ 18.85)}$= 10.15, p= 0.0190, p_{adjust} =0.4794	13
**1E**	CG5 0.05 vs. Veh	$F_{press\ (1.822,\ 18.22)}$= 31.91, p < 0.001, p_{adjust} =< ****0.0001	$F_{rew\ (1.835,\ 18.35)}$= 31.96, p < 0.0001, p_{adjust} =< ****0.0001	$F_{chow\ (1.570,\ 15.70)}$= 12.48, p= 0.0010, p_{adjust} =0.0516	11
**1E**	CGS 0.05 + GBR 7 vs. Veh	$F_{press\ (1.822,\ 18.22)}$= 31.91, p < 0.001, p_{adjust} =< ****0.0001	$F_{rew\ (1.835,\ 18.35)}$= 31.96, p < 0.0001, p_{adjust} =< ****0.0001	$F_{chow\ (1.570,\ 15.70)}$= 12.48, p= 0.0010, p_{adjust} =**0.0055	11
**1E**	CGS 0.05 + GBR 7 vs. CGS + Veh	$F_{press\ (1.822,\ 18.22)}$= 31.91, p < 0.001, p_{adjust} =0.9467	$F_{rew\ (1.835,\ 18.35)}$= 31.96, p < 0.0001, p_{adjust} =0.9645	$F_{chow\ (1.570,\ 15.70)}$= 12.48, p= 0.0010, p_{adjust} =*0.0281	11
**1E**	CGS 0.05 + GBR 7 vs. CGS + Veh	$F_{press\ (1.822,\ 18.22)}$= 31.91, p < 0.001, p_{adjust} =0.9467	$F_{rew\ (1.835,\ 18.35)}$= 31.96, p < 0.0001, p_{adjust} =0.9645	$F_{chow\ (1.570,\ 15.70)}$= 12.48, p= 0.0010, p_{adjust} =*0.0281	11
**1F**	TBZ 0.25 vs. Veh	$F_{press\ (1.014,\ 13.18)}$= 26.43, p= 0.0002, p_{adjust} =0.0842	$F_{rew\ (2.240,\ 29.12)}$= 110.7, p < 0.0001, p_{adjust} =0.0897	$F_{chow\ (2.240,\ 29.12)}$= 11.79, p= 0.0006, p_{adjust} =0.9988	14
**1F**	TBZ 0.5 vs. Veh	$F_{press\ (1.014,\ 13.18)}$= 26.43, p= 0.0002, p_{adjust} =****0.0001	$F_{rew\ (2.240,\ 29.12)}$= 110.7, p < 0.0001, p_{adjust} =**0.0030	$F_{chow\ (2.240,\ 29.12)}$= 11.79, p= 0.0006, p_{adjust} =0.2977	14
**1F**	TBZ 0.75 vs. Veh	$F_{press\ (1.014,\ 13.18)}$= 26.43, p= 0.0002, p_{adjust} =***0.0005	$F_{rew\ (2.240,\ 29.12)}$= 110.7, p < 0.0001, p_{adjust} =**0.0047	$F_{chow\ (2.240,\ 29.12)}$= 11.79, p= 0.0006, p_{adjust} =0.9115	14
**1F**	GBR 10 vs. Veh	$F_{press\ (1.014,\ 13.18)}$= 26.43, p= 0.0002, p_{adjust} =***0.0010	$F_{rew\ (2.240,\ 29.12)}$= 110.7, p < 0.0001, p_{adjust} =< ****0.0001	$F_{chow\ (2.240,\ 29.12)}$= 11.79, p= 0.0006, p_{adjust} =**0.0017	14
**1G**	CAF 10 vs. Veh	$F_{press\ (1.297,\ 15.57)}$= 20.37, p= 0.0002, p_{adjust} =< ****0.0001	$F_{rew\ (2.482,\ 29.78)}$= 36.08, p < 0.0001, p_{adjust} =***0.0002	$F_{chow\ (1.548,\ 18.57)}$= 35.75, p < 0.0001, p_{adjust} =0.1789	13
**1G**	GBR 2.5 vs. Veh	$F_{press\ (1.297,\ 15.57)}$= 20.37, p= 0.0002, p_{adjust} =*0.0497	$F_{rew\ (2.482,\ 29.78)}$= 36.08, p < 0.0001, p_{adjust} =0.0901	$F_{chow\ (1.548,\ 18.57)}$= 35.75, p < 0.0001, p_{adjust} =0.1074	13
**1G**	GBR 7.5 vs. Veh	$F_{press\ (1.297,\ 15.57)}$= 20.37, p= 0.0002, p_{adjust} =**0.0031	$F_{rew\ (2.482,\ 29.78)}$= 36.08, p < 0.0001, p_{adjust} =< ****0.0001	$F_{chow\ (1.548,\ 18.57)}$= 35.75, p < 0.0001, p_{adjust} =**0.0013	13
**1G**	GBR 10 vs. Veh	$F_{press\ (1.297,\ 15.57)}$= 20.37, p= 0.0002, p_{adjust} =***0.0009	$F_{rew\ (2.482,\ 29.78)}$= 36.08, p < 0.0001, p_{adjust} =< ****0.0001	$F_{chow\ (1.548,\ 18.57)}$= 35.75, p < 0.0001, p_{adjust} =< ****0.0001	13

Statistical Analysis for Figures 1A–1G. Statistical analyses for Figures 1A–1G were conducted using repeated measures one-way ANOVA. For Figures 1A, 1B, 1F, and 1G, post-hoc comparisons were corrected using Dunnett’s test. For Figures 1C, 1D, and 1E, Tukey’s test was applied to correct for multiple comparisons.

Supplemental Table 6 (S6): Figures 1H–1K

Fig	Comparison(mg/kg)	Interaction, Treatment and Time Bins or Latency (Lever press)	N
**1H**	TBZ 0.25 vs. Veh (rate)	$F_{inter\ (3.1759,\ 41.2870)}$= 0.65, p_{adjust} =0.615, $F_{treat\ (2.53,\ 32.83)}$= 4.58, p_{adjust} =0.012; $F_{bins\ (1.088,14.15)}$= 4.36, p_{adjust} =0.05; $p_{binadjust}$= 0.9997, 0.2099, 0.9862, 0.8236, 0.9535	14
**1H**	TBZ 0.5 vs. Veh (rate)	$F_{inter\ (3.1759,\ 41.2870)}$= 0.65, p_{adjust} =0.615, $F_{treat\ (2.53,\ 32.83)}$= 4.58, p_{adjust} =0.012; $F_{bins\ (1.088,14.15)}$= 4.36, p_{adjust} =0.05; $p_{binsadjust}$= 0.0668, *0.0423, 0.2802, 0.2529, 0.2998	14
**1H**	TBZ 0.75 vs. Veh (rate)	$F_{inter\ (3.1759,\ 41.2870)}$= 0.65, p_{adjust} =0.615, $F_{treat\ (2.53,\ 32.83)}$= 4.58, p_{adjust} =0.012; $F_{bins\ (1.088,14.15)}$= 4.36, p_{adjust} =0.05; $p_{binsadjust}$= 0.1594, *0.0454, 0.4151, 0.4291, 0.4350	14
**1I**	GBR 2.5 vs. Veh (rate)	$F_{inter\ (5.2664,\ 78.9963)}$= 1.66, p_{adjust} =0.1758, $F_{treat\ (1.77,\ 22.91)}$= 11.95, p_{adjust} =0.007; $F_{bins\ (1.11,\ 14.43)}$= 15.83, p_{adjust} =0.0010, $p_{binsadjust}$= 0.9994, 1.0000, 0.9912, 0.8507, 0.9772	13
**1I**	GBR 7.5 vs. Veh (rate)	$F_{inter\ (5.2664,\ 78.9963)}$= 1.66, p_{adjust} =0.1758, $F_{treat\ (1.77,\ 22.91)}$= 11.95, p_{adjust} =0.007; $F_{bins\ (1.11,\ 14.43)}$= 15.83, p_{adjust} =0.0010, $p_{binsadjust}$= 0.1871, 0.1649, *0.0107, ***0.0009, *0.0175	13
**1I**	GBR 10 vs. Veh (rate)	$F_{inter\ (5.2664,\ 78.9963)}$= 1.66, p_{adjust} =0.1758, $F_{treat\ (1.77,\ 22.91)}$= 11.95, p_{adjust} =0.007; $F_{bins\ (1.11,\ 14.43)}$= 15.83, p_{adjust} =0.0010, $p_{binsadjust}$= **0.0036, **0.0014, ****<0.0001 (bins 3,4), **0.0051	13
**1J**	TBZ 0.25 vs. Veh (lat)	$F_{lat\ (1.7,\ 22.33)}$= 25.3, p= 0.0008, p_{adjust} =0.3200	14
**1J**	TBZ 0.5 vs. Veh (lat)	$F_{lat\ (1.7,\ 22.33)}$= 25.3, p= 0.0008, p_{adjust} =**0.0026	14
**1J**	TBZ 0.75 vs. Veh (lat)	$F_{lat\ (1.7,\ 22.33)}$= 25.3, p= 0.0008, p_{adjust} =**0.0042	14
**1J**	GBR 2.5 vs. Veh (lat)	$F_{lat\ (2.7,\ 35.07)}$= 25.13, p < ****0.0001, p_{adjust} =0.3658	13
**1J**	GBR 7.5 vs. Veh (lat)	$F_{lat\ (2.7,\ 35.07)}$= 25.13, p < ****0.0001, p_{adjust} =< ****0.0001	13
**1J**	GBR 10 vs. Veh (lat)	$F_{lat\ (2.7,\ 35.07)}$= 25.13, p < ****0.0001, p_{adjust} =< ****0.0001	13

Fig	Comparison(mg/kg)	Lever press	Reward	Chow consumption	N
**1K**	AMPH 0.5 vs. Veh	$F_{press\ (1.546,\ 17.01)}$= 9.529, p= 0.0029, p_{adjust} =*0.0129	$F_{rew\ (2.091,\ 23.00)}$= 19.11, p < 0.0001, p_{adjust} =***0.0003	not available	12
**1K**	AMPH 1 vs. Veh	$F_{press\ (1.546,\ 17.01)}$= 9.529, p= 0.0029, p_{adjust} =*0.0142	$F_{rew\ (2.091,\ 23.00)}$= 19.11, p < 0.0001, p_{adjust} =***0.0002	not available	12
**1K**	AMPH 1.5 vs. Veh	$F_{press\ (1.546,\ 17.01)}$= 9.529, p= 0.0029, p_{adjust} =*0.0115	$F_{rew\ (2.091,\ 23.00)}$= 19.11, p < 0.0001, p_{adjust} =< ****0.0001	not available	12
**1L**	CAF 10 vs. Veh	$F_{press\ (1.424,\ 17.08)}$= 24.74, p < 0.0001, p_{adjust} =***0.0001	$F_{rew\ (2.531,\ 30.37)}$= 43.87, p < 0.0001, p_{adjust} =< ****0.0001	$F_{chow\ (2.709,\ 32.51)}$= 74.18, p < 0.0001, p_{adjust} =< ****0.0001	13
**1L**	MOD 10 vs. Veh	$F_{press\ (1.424,\ 17.08)}$= 24.74, p < 0.0001, p_{adjust} =0.0594	$F_{rew\ (2.531,\ 30.37)}$= 43.87, p < 0.0001, p_{adjust} =0.0743	$F_{chow\ (2.709,\ 32.51)}$= 74.18, p < 0.0001, p_{adjust} =0.9343	13
**1L**	MOD 30 vs. Veh	$F_{press\ (1.424,\ 17.08)}$= 24.74, p < 0.0001, p_{adjust} =***0.0001	$F_{rew\ (2.531,\ 30.37)}$= 43.87, p < 0.0001, p_{adjust} =< ****0.0001	$F_{chow\ (2.709,\ 32.51)}$= 74.18, p < 0.0001, p_{adjust} =*0.0185	13
**1L**	MOD 90 vs. Veh	$F_{press\ (1.424,\ 17.08)}$= 24.74, p < 0.0001, p_{adjust} =***0.0005	$F_{rew\ (2.531,\ 30.37)}$= 43.87, p < 0.0001, p_{adjust} =< ****0.0001	$F_{chow\ (2.709,\ 32.51)}$= 74.18, p < 0.0001, p_{adjust} =< ****0.0001	13

Statistical Analysis for Figures 1H–1L: Figures 1H-1I were analyzed using repeated measures two-way ANOVA, while 1J was analyzed using repeated measures one-way ANOVA. For 1K, due to some missing data, an ordinary one-way ANOVA was applied instead of repeated measures. For all rows, multiple comparisons were corrected using Dunnett's test.

Supplemental Table 7 (S7): Figures 2A–2F

Fig	Comparison$_{(mg/kg)}$	Screen touch	Reward	Chow consumption	N
**2A**	TBZ 2.5 vs. Veh	$F_{touch\ (3.396,\ 40.75)}$= 2.733, p= 0.0498, p_{adjust} =0.214	$F_{rew\ (3.064,\ 36.77)}$= 5.304, p= 0.0037, p_{adjust} =0.0815	$F_{chow\ (3.034,\ 36.41)}$= 0.7583, p= 0.5262, p_{adjust} =0.3757	13
**2A**	TBZ 7.5 vs. Veh	$F_{touch\ (3.396,\ 40.75)}$= 2.733, p= 0.0498, p_{adjust} =0.1834	$F_{rew\ (3.064,\ 36.77)}$= 5.304, p= 0.0037, p_{adjust} =* 0.0427	$F_{chow\ (3.034,\ 36.41)}$= 0.7583, p= 0.5262, p_{adjust} =0.8019	13
**2A**	GBR 3 vs. Veh	$F_{touch\ (3.396,\ 40.75)}$= 2.733, p= 0.0498, p_{adjust} =0.4706	$F_{rew\ (3.064,\ 36.77)}$= 5.304, p= 0.0037, p_{adjust} =0.5238	$F_{chow\ (3.034,\ 36.41)}$= 0.7583, p= 0.5262, p_{adjust} =0.7922	13
**2A**	GBR 10 vs. Veh	$F_{touch\ (3.396,\ 40.75)}$= 2.733, p= 0.0498, p_{adjust} =0.994	$F_{rew\ (3.064,\ 36.77)}$= 5.304, p= 0.0037, p_{adjust} =0.1717	$F_{chow\ (3.034,\ 36.41)}$= 0.7583, p= 0.5262, p_{adjust} =0.9994	13
**2B**	HP 0.1 vs. Veh	$F_{touch\ (2.647,\ 37.06)}$= 6.080, p= 0.0026, p_{adjust} =0.2217	$F_{rew\ (2.606,\ 36.48)}$= 7.652, p= 0.0007, p_{adjust} =0.0655	$F_{chow\ (2.019,\ 28.27)}$= 4.036, p= 0.0284, p_{adjust} =0.2461	15
**2B**	TBZ 10 vs. Veh	$F_{touch\ (2.647,\ 37.06)}$= 6.080, p= 0.0026, p_{adjust} =*0.0299	$F_{rew\ (2.606,\ 36.48)}$= 7.652, p= 0.0007, p_{adjust} =*0.0139	$F_{chow\ (2.019,\ 28.27)}$= 4.036, p= 0.0284, p_{adjust} =0.2369	15
**2B**	GBR 10 vs. Veh	$F_{touch\ (2.647,\ 37.06)}$= 6.080, p= 0.0026, p_{adjust} =0.5866	$F_{rew\ (2.606,\ 36.48)}$= 7.652, p= 0.0007, p_{adjust} =0.6659	$F_{chow\ (2.019,\ 28.27)}$= 4.036, p= 0.0284, p_{adjust} =***0.0005	15
**2C**	TBZ 7.5 vs. Veh	$F_{touch\ (1.148,\ 16.07)}$= 5.468, p= 0.0288, p_{adjust} =0.3212	$F_{rew\ (4,\ 70)}$= 1.419, p= 0.2367, p_{adjust} =0.1041	$F_{chow\ (2.666,\ 37.32)}$= 1.899, p= 0.1522, p_{adjust} =0.991	15
**2C**	TBZ 10 vs. Veh	$F_{touch\ (1.148,\ 16.07)}$= 5.468, p= 0.0288, p_{adjust} =0.2535	$F_{rew\ (4,\ 70)}$= 1.419, p= 0.2367, p_{adjust} =0.3301	$F_{chow\ (2.666,\ 37.32)}$= 1.899, p= 0.1522, p_{adjust} =0.9318	15
**2C**	GBR 3 vs. Veh	$F_{touch\ (1.148,\ 16.07)}$= 5.468, p= 0.0288, p_{adjust} =0.9933	$F_{rew\ (4,\ 70)}$= 1.419, p= 0.2367, p_{adjust} =0.7794	$F_{chow\ (2.666,\ 37.32)}$= 1.899, p= 0.1522, p_{adjust} =0.4261	15
**2C**	GBR 10 vs. Veh	$F_{touch\ (1.148,\ 16.07)}$= 5.468, p= 0.0288, p_{adjust} =0.1617	$F_{rew\ (4,\ 70)}$= 1.419, p= 0.2367, p_{adjust} =0.9128	$F_{chow\ (2.666,\ 37.32)}$= 1.899, p= 0.1522, p_{adjust} =0.0779	15

Fig	Comparison$_{(mg/kg)}$	Interaction, Treatment and Time Bins or Latency (lat)	N
**2D**	GBR 3 vs. Veh (rate)	$F_{inter\ (8,\ 112)}$= 0.8378, p_{adjust} =0.5714, $F_{treat\ (4,56)}$= 5.3504, p_{adjust} =0.0010, $F_{bins\ (2,28)}$= 19.1958, p_{adjust} =0.0001, $p_{binadjust}$= 0.9998, 0.9971, 0.9951, 0.9924, 1.0000	15
**2D**	GBR 10 vs. Veh (rate)	$F_{inter\ (8,\ 112)}$= 0.8378, p_{adjust} =0.5714, $F_{treat\ (4,56)}$= 5.3504, p_{adjust} =0.0010, $F_{bins\ (2,28)}$= 19.1958, p_{adjust} =0.0001, $p_{binadjust}$= **0.0051, **0.0028, **0.0025, *0.0271, *0.0244	15
**2D**	TBZ 7.5 vs. Veh (rate)	$F_{inter\ (8,\ 112)}$= 0.8378, p_{adjust} =0.5714, $F_{treat\ (4,56)}$= 5.3504, p_{adjust} =0.0010, $F_{bins\ (2,28)}$= 19.1958, p_{adjust} =0.0001, $p_{binadjust}$= 1.0000, 0.9803, 0.9971, 0.9857, 0.9801	15
**2D**	TBZ 10 vs. Veh (rate)	$F_{inter\ (8,\ 112)}$= 0.8378, p_{adjust} =0.5714, $F_{treat\ (4,56)}$= 5.3504, p_{adjust} =0.0010, $F_{bins\ (2,28)}$= 19.1958, p_{adjust} =0.0001, $p_{binadjust}$= 0.9998, 1.0000, 0.9984, 0.9760, 0.7816	15
**2D**	GBR 3 vs. Veh (lat)	$F_{lat\ (3.0875,\ 43.23)}$= 2.8759, p= 0.0456, p_{adjust} =0.8578	15
**2D**	GBR 10 vs. Veh (lat)	$F_{lat\ (3.0875,\ 43.23)}$= 2.8759, p= 0.0456, p_{adjust} =1.0000	15
**2D**	TBZ 7.5 vs. Veh (lat)	$F_{lat\ (3.0875,\ 43.23)}$= 2.8759, p= 0.0456, p_{adjust} =0.1560	15
**2D**	TBZ 10 vs. Veh (lat)	$F_{lat\ (3.0875,\ 43.23)}$= 2.8759, p= 0.0456, p_{adjust} =0.4876	15

Fig	Comparison$_{(mg/kg)}$	Screen touch	Reward	Chow consumption	N
**2E**	HP 0.1 vs. Veh	$F_{touch\ (2.482,\ 34.75)}$= 2.519, p= 0.0842, p_{adjust} =0.2395	$F_{rew\ (2.174,\ 30.43)}$= 5.774, p= 0.0063, p_{adjust} =** 0.0069	$F_{chow\ (2.818,\ 39.45)}$= 1.439, p= 0.2471, p_{adjust} =0.5428	15
**2E**	TBZ 10 vs. Veh	$F_{touch\ (2.482,\ 34.75)}$= 2.519, p= 0.0842, p_{adjust} =0.2539	$F_{rew\ (2.174,\ 30.43)}$= 5.774, p= 0.0063, p_{adjust} =* 0.0498	$F_{chow\ (2.818,\ 39.45)}$= 1.439, p= 0.2471, p_{adjust} =0.1691	15
**2E**	MPH 3 vs. Veh	$F_{touch\ (2.482,\ 34.75)}$= 2.519, p= 0.0842, p_{adjust} =0.6117	$F_{rew\ (2.174,\ 30.43)}$= 5.774, p= 0.0063, p_{adjust} =0.7051	$F_{chow\ (2.818,\ 39.45)}$= 1.439, p= 0.2471, p_{adjust} =0.7324	15
**2E**	AMPH 0.3 vs. Veh	$F_{touch\ (2.482,\ 34.75)}$= 2.519, p= 0.0842, p_{adjust} => 0.9999	$F_{rew\ (2.174,\ 30.43)}$= 5.774, p= 0.0063, p_{adjust} =0.7025	$F_{chow\ (2.818,\ 39.45)}$= 1.439, p= 0.2471, p_{adjust} =0.3885	15
**2F**	HP 0.1 vs. Veh	$F_{touch(1.384,\ 16.61)}$= 46.75, p <0.0001, p_{adjust} =*** 0.0001	$F_{rew\ (1.919,\ 23.02)}$= 20.06, p <0.0001, p_{adjust} =*** 0.0004	$F_{chow\ (1.843,\ 27.65)}$= 5.124, p= 0.0146, p_{adjust} =0.7926	13
**2F**	TBZ 10 vs. Veh	$F_{touch(1.384,\ 16.61)}$= 46.75, p <0.0001, p_{adjust} =0.2865	$F_{rew\ (1.919,\ 23.02)}$= 20.06, p <0.0001, p_{adjust} =0.2165	$F_{chow\ (1.843,\ 27.65)}$= 5.124, p= 0.0146, p_{adjust} =0.3361	13
**2F**	GBR 10 vs. Veh	$F_{touch(1.384,\ 16.61)}$= 46.75, p <0.0001, p_{adjust} =*** 0.0001	$F_{rew\ (1.919,\ 23.02)}$= 20.06, p <0.0001, p_{adjust} =** 0.0084	$F_{chow\ (1.843,\ 27.65)}$= 5.124, p= 0.0146, p_{adjust} =* 0.0135	13

Statistical Analysis for Figures 2A–2F: Figures 2A–2C and 2D (latency)–2F were analyzed using repeated measures one-way ANOVA, while Figure 2D (rate) was analyzed using repeated measures two-way ANOVA. For all panels, Dunnett's test was used to correct for multiple comparisons.

Supplemental Table 8 (S8): Figure 2G-2I

Fig	Comparison(mg/kg)	Screen touch	Reward	Chow consumption	N
2G	CGS 0.01 vs. Veh	$F_{touch\ (2.389,\ 38.23)} = 25.52$, $p < 0.0001$, $p_{adjust} = 0.8433$	$F_{rew\ (1.863,\ 29.81)} = 29.74$, $p < 0.0001$, $p_{adjust} = 0.1607$	$F_{chow\ (2.127,\ 34.04)} = 21.63$, $p < 0.0001$, p_{adjust} = ***0.0007	17
2G	CGS 0.05 vs. Veh	$F_{touch\ (2.389,\ 38.23)} = 25.52$, $p < 0.0001$, p_{adjust} = ** 0.0091	$F_{rew\ (1.863,\ 29.81)} = 29.74$, $p < 0.0001$, p_{adjust} = ** 0.0067	$F_{chow\ (2.127,\ 34.04)} = 21.63$, $p < 0.0001$, p_{adjust} < ****0.0001	17
2G	CGS 0.2 vs. Veh	$F_{touch\ (2.389,\ 38.23)} = 25.52$, $p < 0.0001$, p_{adjust} < ****0.0001	$F_{rew\ (1.863,\ 29.81)} = 29.74$, $p < 0.0001$, p_{adjust} **** < 0.0001	$F_{chow\ (2.127,\ 34.04)} = 21.63$, $p < 0.0001$, p_{adjust} = ***0.0002	17
**Fig**	**Comparison(mg/kg)**	**Interaction, Treatment and Time Bins or Latency (Screen touch)**			**N**
2H	HP 0.1 vs. Veh (rate)	$F_{inter\ (3.3537,\ 40.24)} = 9.9459$, $p_{adjust} = <0.0001$, $F_{treat\ (1.8878,\ 22.65)} = 27.48$, $p_{adjust} = <0.000$, $F_{bins\ (2.5171,\ 30.20)} = 38.5028$, $p_{adjust} = <0.0001$, $p_{adjustbin}$ = 0.9999, 0.6717, 0.2914, 0.0971, 0.0518			13
2H	TBZ 10 vs. Veh (rate)	$F_{inter\ (3.3537,\ 40.24)} = 9.9459$, $p_{adjust} = <0.0001$, $F_{treat\ (1.8878,\ 22.65)} = 27.48$, $p_{adjust} = <0.000$, $F_{bins\ (2.5171,\ 30.20)} = 38.5028$, $p_{adjust} = <0.0001$, $p_{adjustbin}$ = 0.9998, 0.9279, 0.9998, 0.7156, 0.8067			13
2H	GBR 10 vs. Veh (rate)	$F_{inter\ (3.3537,\ 40.24)} = 9.9459$, $p_{adjust} = <0.0001$, $F_{treat\ (1.8878,\ 22.65)} = 27.48$, $p_{adjust} = <0.0001$, $F_{bins\ (2.5171,\ 30.20)} = 38.5028$, $p_{adjust} = <0.0001$, $p_{adjustbin}$=***0.0003, <****0.0001 (bins 2-4),***0.0002			13
2I	CGS 0.01 vs. Veh (rate)	$F_{inter\ (4.0668,\ 65.07)} = 3.5417$, $p_{adjust} = 0.0108$, $F_{treat\ (2.1095,\ 33.75)} = 6.3391$, $p_{adjust} = 0.0041$, $F_{bins\ (1.2365,\ 19.78)} = 17.3432$, $p_{adjust} = 0.0002$, $p_{binadjust}$ = 0.9159, 0.5849, 0.5253, 0.9380, 0.9894			17
2I	CGS 0.05 vs. Veh (rate)	$F_{inter\ (4.0668,\ 65.07)} = 3.5417$, $p_{adjust} = 0.0108$, $F_{treat\ (2.1095,\ 33.75)} = 6.3391$, $p_{adjust} = 0.0041$, $F_{bins\ (1.2365,\ 19.78)} = 17.3432$, $p_{adjust} = 0.0002$, $p_{binadjust}$ = 0.4819, 0.9400, 0.9398, 0.4419, 0.6074			17
2I	CGS 0.2 vs. Veh (rate)	$F_{inter\ (4.0668,\ 65.07)} = 3.5417$, $p_{adjust} = 0.0108$, $F_{treat\ (2.1095,\ 33.75)} = 6.3391$, $p_{adjust} = 0.0041$, $F_{bins\ (1.2365,\ 19.78)} = 17.3432$, $p_{adjust} = 0.0002$, $p_{binadjust}$ = 0.3904, 0.6999, 0.9606, 0.6671, 0.3218			17
2J	HP 0.1 vs. Veh (lat)	$F_{lat\ (2.2620,\ 27.14)} = 15.2457$, $p < 0.0001$, $p_{adjust} = 0.1308$			13
2J	TBZ 10 vs. Veh (lat)	$F_{lat\ (2.2620,\ 27.14)} = 15.2457$, $p < 0.0001$, $p_{adjust} = 0.6398$			13
2J	GBR 10 vs. Veh (lat)	$F_{lat\ (2.2620,\ 27.14)} = 15.2457$, $p < 0.0001$, $p_{adjust} = 0.0585$			13
2J	CGS 0.01 vs. Veh (lat)	$F_{lat\ (1.3979,\ 22.37)} = 4.7984$, $p = 0.0019$, $p_{adjust} = 1.0000$			17
2J	CGS 0.05 vs. Veh (lat)	$F_{lat\ (1.3979,\ 22.37)} = 4.7984$, $p = 0.0019$, $p_{adjust} = 1.0000$			17
2J	CGS 0.20 vs. Veh (lat)	$F_{lat\ (1.3979,\ 22.37)} = 4.7984$, $p = 0.0019$, p_{adjust} =**0.0018			17

Statistical Analysis for Figures 2G–2I: Figures 2H-2I were analyzed using repeated measures two-way ANOVA, while Figures 2G and 2J were analyzed using repeated measures one-way ANOVA. For all panels, Dunnett's test was applied to correct for multiple comparisons.

Supplemental Table 9 (S9): Figure 3

Fig	Comparison$_{(mg/Kg)}$	Lever / Screen touch	Rewards	Chow consumption	N
**3A**	Day vs. Lever Press	$F_{press\ (2.051,\ 28.71)}$= 25.12, p < ****0.0001	not available	not available	15
**3A**	GBR 10 vs. Veh	T(14)= 1.432,p= 0.1741	not available	t(14)= 4.907, p= ***0.0002	15
**3B**	Rat sERC vs. mERC	not available	not available	t(10)= 6.129, p= ***0.0001	11-13
**3B**	Mouse sERC vs. mERC	not available	not available	t(27)=6.419, p < ****0.0001	13-16
**3C**	Veh vs. Veh (DEV)	$F_{press\ (1.933,\ 19.33)}$= 14.85, p= 0.0001, p_{adjust} =*0.0118	$F_{rews\ (1.928,\ 19.28)}$= 16.08, p < 0.0001, p_{adjust} =**0.0084	$F_{chow\ (1.070,\ 10.70)}$= 97.98, p < 0.0001, p_{adj} < ****0.0001	11
**3C**	GBR 7.5 (DEV) vs. Veh (DEV)	$F_{press\ (1.933,\ 19.33)}$= 14.85, p= 0.0001, p_{adjust} =***0.0005	$F_{rews\ (1.928,\ 19.28)}$= 16.08, p < 0.0001, p_{adjust} =***0.0005	$F_{chow\ (1.070,\ 10.70)}$= 97.98, p < 0.0001, p_{adjust} < ****0.0001	11
**3C**	GBR_7.5(DEV) vs. Veh (DEV)	$F_{press\ (1.933,\ 19.33)}$= 14.85, p= 0.0001, p_{adjust} =0.4133	$F_{rews\ (1.928,\ 19.28)}$= 16.08, p < 0.0001, p_{adjust} =0.4367	$F_{chow\ (1.070,\ 10.70)}$= 97.98, p < 0.0001, p_{adjust} =0.2098	11
**3D**	HP 0.1 vs. Veh	$F_{touch\ (1.597,\ 22.35)}$= 22.84, p <0.0001, p_{adjust} =< ****0.0001	$F_{rews\ (1.803,\ 25.24)}$= 11.69, p= 0.0004, p_{adjust} =*** 0.0009	no chow choice	15
**3D**	GBR 10 vs. Veh	$F_{touch\ (1.597,\ 22.35)}$= 22.84, p <0.0001, p_{adjust} =0.2399	$F_{rews\ (1.803,\ 25.24)}$= 11.69, p= 0.0004, p_{adjust} =0.8065	no chow choice	15
**Fig**	**Comparison$_{(mg/Kg)}$**	**Interaction, Treatment and Time Bins or Latency (Screen touch)**			**N**
**3E**	HP 0.1 vs. Veh (rate)	$F_{inter\ (3.3877,\ 50.81)}$= 9.6838, p_{adjust} =< 0.0001, $F_{treat\ (1.6400,\ 24.6003)}$= 33.1253, p_{adjust} =< 0.0001, $F_{bins\ (1.6310,\ 24.46)}$= 49.6078, p_{adjust} =< 0.0001, $p_{binadjust}$= 0.4169, 0.0074, **0.0018, < ****0.0001(bins 4,5)			15
**3E**	GBR 10 vs. Veh (rate)	$F_{inter\ (3.3877,\ 50.81)}$= 9.6838, p_{adjust} =< 0.0001, $F_{treat\ (1.6400,\ 24.6003)}$= 33.1253, p_{adjust} =< 0.0001, $F_{bins\ (1.6310,\ 24.46)}$= 49.6078, p_{adjust} =< 0.0001, $p_{binadjust}$= **0.0065, 0.4816, **0.0054, 0.9671, 0.8837			15
**3E**	HP 0.1 vs. Veh (lat)	$F_{lat\ (1.9717,\ 29.57)}$= 18.6343, p < 0.0001, p_{adjust} =***0.0005			15
**3E**	GBR 10 vs. Veh (lat)	$F_{lat\ (1.9717,\ 29.57)}$= 18.6343, p < 0.0001, p_{adjust} =0.3164			15

Statistical Analysis for Figure 3: Figures 3A-3B were analyzed using paired t-tests; 3F with unpaired t-test. One-way ANOVA was applied to 3A, 3C–3D, 3E (latency); two-way ANOVA to 3E (rate) and 3F. No multiple comparisons correction was applied to 3A–3B and 3F (t-test). Post-hoc corrections included Tukey's (3C), Sidak's (3F ANOVA), and Dunnett's test (all others).

Supplemental Table 10 (S10): Figure 4

Fig	Comparison(mg/Kg)	Interaction, Treatment and Time Bins or Latency (Screen touch)	N
4A	TBZ 1.5 + Veh vs. Veh	$p_{inter} < 0.0001$, $p_{treat} = 0.0003$, $p_{time} < 0.0001$, $p_{binadjust} =$ 0.965, 0.0518, *0.0218, ***0.0002, ***0.0003, ***0.0005, **0.0025, **0.0023, *0.0434, **0.0064, *0.0122, **0.0040, *0.0122	9-10 /grp
4A	Veh + GBR 10	$p_{inter} < 0.0001$, $p_{treat} = 0.0003$, $p_{time} < 0.0001$, $p_{binadjust} =$ 0.9885, 0.4977, 0.4418, 0.5036, 0.3003, 0.2639, 0.1885, 0.0824, 0.0572, *0.0333, *0.0137, **0.0097, **0.0081	9-10 /grp
4A	TBZ 1.5 + GBR 10 vs. Veh	$p_{inter} < 0.0001$, $p_{treat} = 0.0003$, $p_{time} < 0.0001$, $p_{binadjust} =$ 0.4975, 0.1271, 0.5194, **0.0011, ***0.0006, 0.0906, **0.0083, **0.0043, *0.0192, *0.0492, *0.0203, *0.0151, *0.0152	9-10 /grp
4B	TBZ 1.5 + Veh vs. Veh	$p_{inter} < 0.0001$, $p_{treat} = 0.0018$, $p_{time} = 0.0001$, $p_{binadjust} =$ 0.4291, ***0.0003, *0.0180, ***0.0003, <****0.0001 (bins 5,6), **0.0028, *0.0209, 0.0848, **0.0047, **0.0014, *0.0282, *0.0110	9-10 /grp
4B	Veh + GBR 10 vs. Veh	$p_{inter} < 0.0001$, $p_{treat} = 0.0018$, $p_{time} = 0.0001$, $p_{bins\ (1\text{-}13)\ adjust} =$ 0.4412, >0.9999, 0.9982, 0.9017, 0.9743, 0.5229, 0.3019, 0.1970, 0.0539, 0.0800, *0.0450, *0.0289, *0.0280	9-10 /grp
4B	TBZ 1.5 + GBR 10 vs. Veh	$p_{inter} < 0.0001$, $p_{treat} = 0.0018$, $p_{time} = 0.0001$, $p_{binadjust} =$ 0.4605, ***0.0002, 0.8667, ***0.0005, <****0.0001, 0.1565, ***0.0009, **0.0042, **0.0070, **0.0059, **0.0060, *0.0192, **0.0060	9-10 /grp

Fig	Comparison(mg/Kg)	Lever or receptacle entry	Rewards / cues	Chow consumption	N
4D	TBZ 0.75 vs. Veh	$F_{press\ (2.085,\ 14.60)} = 29.90$, $p < 0.0001$, $p_{adjust} =$ **0.0043	$F_{rew\ (2.477,\ 17.34)} = 32.32$, $p < 0.001$, $p_{adjust} =$ **0.0012	$F_{chow\ (2.184,\ 15.29)} = 5.70$, $p = 0.0126$, $p_{adjust} = 0.6403$	8
4D	GBR 2.5 vs. Veh	$F_{press\ (2.085,\ 14.60)} = 29.90$, $p < 0.0001$, $p_{adjust} = 0.0909$	$F_{rew\ (2.477,\ 17.34)} = 32.32$, $p < 0.001$, $p_{adjust} = 0.2718$	$F_{chow\ (2.184,\ 15.29)} = 5.70$, $p = 0.0126$, $p_{adjust} = 0.9989$	8
4D	GBR 7.5 vs. Veh	$F_{press\ (2.085,\ 14.60)} = 29.90$, $p < 0.0001$, $p_{adjust} =$ **0.0032	$F_{rew\ (2.477,\ 17.34)} = 32.32$, $p < 0.001$, $p_{adjust} =$ **0.0070	$F_{chow\ (2.184,\ 15.29)} = 5.70$, $p = 0.0126$, $p_{adj} =$ *0.0307	8
4E	TBZ 0.75 vs. Veh	$F_{lever\ (1.642,\ 11.49)} = 1.597$, $p = 0.2426$, $p_{adjust} = 0.1737$	$F_{cue\ (1.776,\ 12.43)} = 10.51$, $p = 0.0026$, $p_{adjust} =$ *0.0296	see above	8
4E	TBZ 0.75 vs. Veh	$F_{recep\ (1.300,\ 9.097)} = 3.972$, $p = 0.0701$, $p_{adjust} = 0.7436$	$F_{rew\ (2.070,\ 14.49)} = 1.443$, $p = 0.2687$, $p_{adjust} = 0.9714$	see above	8
4F	GBR 2.5 vs. Veh	$F_{lever\ (1.642,\ 11.49)} = 1.597$, $p = 0.2426$, $p_{adjust} = 0.7299$	$F_{cue\ (1.776,\ 12.43)} = 10.51$, $p = 0.0026$, $p_{adj} = 0.9921$	see above	8
4F	GBR 7.5 vs. Veh	$F_{lever\ (1.642,\ 11.49)} = 1.597$, $p = 0.2426$, $p_{adjust} = 0.8925$	$F_{cue\ (1.776,\ 12.43)} = 10.51$, $p = 0.0026$, $p_{adj} =$ *0.0402	see above	8
4F	GBR 2.5 vs. Veh	$F_{recep\ (1.300,\ 9.097)} = 3.972$, $p = 0.0701$, $p_{adjust} =$ **0.0030	$F_{rew\ (2.070,\ 14.49)} = 1.443$, $p = 0.2687$, $p_{adjust} = 0.2942$	see above	8
4F	GBR 7.5 vs. Veh	$F_{recep\ (1.300,\ 9.097)} = 3.972$, $p = 0.0701$, $p_{adjust} = 0.1496$	$F_{rew\ (2.070,\ 14.49)} = 1.443$, $p = 0.2687$, $p_{adjust} = 0.5271$	see above	8

Statistical Analysis for Figure 4: Figures 4A-B were analyzed with mixed models and one-way ANOVA was applied to 4D–4F. Post-hoc corrections with Dunnett's test.

Supplemental Table 11 (S11): Figure 5

Fig	Comparison$_{(mg/kg)}$	Statistics	N
**5B**	TBZ 0.25 vs Veh	F_{treat} = 22.09, p < 0.0001, p_{adjust} = 0.7518	5 ctrl, 6/TBZ group
**5B**	TBZ 0.75 vs Veh	F_{treat} = 22.09, p < 0.0001, p_{adjust} < ****0.0001	5 ctrl, 6/TBZ group
**5B**	TBZ 1.5 vs Veh	F_{treat} = 22.09, p < 0.0001, p_{adjust} < ****0.0001	5 ctrl, 6/TBZ group
**5B**	TBZ dose-response	r^2 = 0.8886, p = 0.0574	5 ctrl, 6/TBZ group
**5C**	TBZ 0.25 vs Veh	F_{treat} = 7.307, p = 0.0009, p_{adjust} = 0.5651	4/group
**5C**	TBZ 0.75 vs Veh	F_{treat} = 7.307, p = 0.0009, p_{adjust} = *0.0356	4/group
**5C**	TBZ 1.5 vs Veh	F_{treat} = 7.307, p = 0.0009, p_{adjust} = ***0.004	4/group
**5C**	TBZ dose-response	r^2 = 0.9745, p = **0.0128	4/group
**5D**	TBZ 2.5 vs Veh	F_{treat} = 6.681, p = 0.0009, p_{adjust} = 0.5537	5 ctrl, 6/TBZ group
**5D**	TBZ 5 vs Veh	F_{treat} = 6.681, p = 0.0009, p_{adjust} = **0.0014	5 ctrl, 6/TBZ group
**5D**	TBZ 10 vs Veh	F_{treat} = 6.681, p = 0.0009, p_{adjust} = **0.0042	5 ctrl, 6/TBZ group
**5D**	TBZ dose-response	r^2 = 0.5919, p = 0.2306	5 ctrl, 6/TBZ group
**5E**	TBZ 2.5 vs Veh	F_{treat} = 6.650, p = 0.0009, p_{adjust} = 0.8503	5 ctrl, 6/TBZ group
**5E**	TBZ 5 vs Veh	F_{treat} = 6.650, p = 0.0009, p_{adjust} = **0.0028	5 ctrl, 6/TBZ group
**5E**	TBZ 10 vs Veh	F_{treat} = 6.650, p = 0.0009, p_{adjust} = **0.0063	5 ctrl, 6/TBZ group
**5E**	TBZ dose-response	r^2 = 0.8165, p = 0.0964	5 ctrl, 6/TBZ group
**5F**	TBZ 0.25 vs Veh	F_{treat} = 22.09, p < 0.0001, p_{adjust} = 0.7518	5 ctrl, 6/TBZ group
**5F**	TBZ 0.75 vs Veh	F_{treat} = 22.09, p < 0.0001, p_{adjust} < ****0.0001	5 ctrl, 6/TBZ group
**5F**	TBZ 1.5 vs Veh	F_{treat} = 22.09, p < 0.0001, p_{adjust} < ****0.0001	5 ctrl, 6/TBZ group
**5F**	TBZ 2.5 vs Veh	F_{treat} = 6.681, p = 0.0009, p_{adjust} = 0.5537	5 ctrl, 6/TBZ group
**5F**	TBZ 5 vs Veh	F_{treat} = 6.681, p = 0.0009, p_{adjust} = **0.0014	5 ctrl, 6/TBZ group
**5F**	TBZ 10 vs Veh	F_{treat} = 6.681, p = 0.0009, p_{adjust} = **0.0042	5 ctrl, 6/TBZ group
**5F**	TBZ 0.25 vs Veh	Hedge's $g_{estimate}$ = -0.266, ci = 0.597, -1.129 (small)	5 ctrl, 6/TBZ group
**5F**	TBZ 0.75 vs Veh	Hedge's $g_{estimate}$ = -2.296, ci = -1.191, -3.401 (large)	5 ctrl, 6/TBZ group
**5F**	TBZ 1.5 vs Veh	Hedge's $g_{estimate}$ = -3.023, ci = -1.739, -4.307 (large)	5 ctrl, 6/TBZ group
**5F**	TBZ 2.5 vs Veh	Hedge's $g_{estimate}$ = -0.407, ci = 0.461, -1.275 (small)	5 ctrl, 6/TBZ group
**5F**	TBZ 5 vs Veh	Hedge's $g_{estimate}$ = -1.850, ci = -0.824, -2.875 (large)	5 ctrl, 6/TBZ group
**5F**	TBZ 10 vs Veh	Hedge's $g_{estimate}$ = -1.216, ci = -0.281, -2.150 (large)	5 ctrl, 6/TBZ group
**5G**	TBZ 0.25 vs Veh	F_{treat} = 7.307, p = 0.0009, p_{adjust} = 0.5651	4/group
**5G**	TBZ 0.75 vs Veh	F_{treat} = 7.307, p = 0.0009, p_{adjust} = *0.0356	4/group
**5G**	TBZ 1.5 vs Veh	F_{treat} = 7.307, p = 0.0009, p_{adjust} = ***0.0004	4/group
**5G**	TBZ 2.5 vs Veh	F_{treat} = 6.650, p = 0.0009, p_{adjust} = 0.8503	5 ctrl, 6/TBZ group
**5G**	TBZ 5 vs Veh	F_{treat} = 6.650, p = 0.0009, p_{adjust} = **0.0028	5 ctrl, 6/TBZ group
**5G**	TBZ 10 vs Veh	F_{treat} = 6.650, p = 0.0009, p_{adjust} = **0.0063	5 ctrl, 6/TBZ group
**5G**	TBZ 0.25 vs Veh	Hedge's $g_{estimate}$ = -0.235, ci = 0.621, -1.098 (small)	4/group
**5G**	TBZ 0.75 vs Veh	Hedge's $g_{estimate}$ = -1.775, ci = -0.762, -2.788 (large)	4/group
**5G**	TBZ 1.5 vs Veh	Hedge's $g_{estimate}$ = -1.291, ci = -0.347, -2.234 (large)	4/group
**5G**	TBZ 2.5 vs Veh	Hedge's $g_{estimate}$ = -0.495, ci = 0.502, -5.130 (small)	5 ctrl, 6/TBZ group
**5G**	TBZ 5 vs Veh	Hedge's $g_{estimate}$ = -1.119, ci = -0.029, -2.209 (large)	5 ctrl, 6/TBZ group
**5G**	TBZ 10 vs Veh	Hedge's $g_{estimate}$ = -2.668, ci = -1.289, -4.086 (large)	5 ctrl, 6/TBZ group
**5H**	rat vs mouse	t(18) = 2.730, p = *0.0137	5 rats, 5 mice
**5H**	rat vs mouse	t(17) = 4.040, p = ***0.0008	4 rats, 5 mice

Statistical Analysis for Figure 5: Statistical analyses were conducted as follows: correlations were assessed in Figures 5B-5E (TBZ dose-response). Hedge's *g* was reported in Figures 5F-5G and unpaired two-tailed *t*-tests were used for Figure 5H. No multiple comparison correction was applied to these analyses. All other comparisons were performed using ordinary one-way ANOVA followed by Dunnett's test to correct for multiple comparisons.

REFERENCES

1. Buck K, Voehringer P, Ferger B. Rapid analysis of GABA and glutamate in microdialysis samples using high performance liquid chromatography and tandem mass spectrometry. J Neurosci Methods. 2009;182:78–84.

2. Wong J-MT, Malec PA, Mabrouk OS, Ro J, Dus M, Kennedy RT. Benzoyl chloride derivatization with liquid chromatography–mass spectrometry for targeted metabolomics of neurochemicals in biological samples. J Chromatogr A. 2016;1446:78–90.

3. Li Y, Liu Z, Guo Q, Luo M. Long-term Fiber Photometry for Neuroscience Studies. Neurosci Bull. 2019;35:425–433.

4. Shariatgorji M, Nilsson A, Fridjonsdottir E, Vallianatou T, Källback P, Katan L, et al. Comprehensive mapping of neurotransmitter networks by MALDI–MS imaging. Nat Methods. 2019;16:1021–1028.

5. Shariatgorji R, Nilsson A, Fridjonsdottir E, Strittmatter N, Dannhorn A, Svenningsson P, et al. Spatial visualization of comprehensive brain neurotransmitter systems and neuroactive substances by selective in situ chemical derivatization mass spectrometry imaging. Nat Protoc. 2021;16:3298–3321.
